# Contact-Time Dependence and Cross-Polarization Kinetics of Apparent Signal Distributions in CP/MAS ^13^C NMR Spectra of Humic Acids

**DOI:** 10.3390/molecules31173044

**Published:** 2026-08-30

**Authors:** Evgeny Lodygin, Roman Vasilevich, Evgeny Abakumov

**Affiliations:** 1 Institute of Biology, Komi Science Center, Ural Branch, Russian Academy of Sciences, 28, Kommunisticheskaya st., Syktyvkar 167982, Russia; vasilevich.r.s@ib.komisc.ru; 2 Department of Applied Ecology, Faculty of Biology, St. Petersburg State University, 16th Liniya V.O., 29, St., Petersburg 199178, Russia; e.abakumov@spbu.ru

**Keywords:** soil organic matter, cross-polarization, peat soils

## Abstract

Humic acids (HAs) represent structurally heterogeneous supramolecular systems whose characterization by CP/MAS ^13^C NMR spectroscopy strongly depends on experimental conditions. Among these, contact time is one of the most important parameters controlling the efficiency of polarization transfer and the relative contribution of individual carbon groups. The aim of this study was to evaluate the influence of contact time on the apparent CP/MAS ^13^C NMR signal distribution of HAs isolated from different horizons of a permafrost peat soil (Hemic Folic Cryic Histosol) and to characterize their cross-polarization dynamics using kinetic modeling. A series of CP/MAS ^13^C NMR spectra was acquired at contact times ranging from 0.01 to 10 ms. Signal intensities of major structural groups were analyzed using both the classical three-parameter model and an extended four-parameter model of cross-polarization dynamics. The results demonstrated pronounced contact-time-dependent variations in the apparent CP/MAS signal distribution among alkyl, aryl, and carboxyl/amide structural domains. Short contact times preferentially enhanced proton-rich aliphatic and carbohydrate structures, whereas longer contact times increased the relative contribution of aromatic and carboxyl/amide fragments. The extended model provided a better fit for selected structurally heterogeneous spectral regions, particularly aromatic and heteroatom-containing domains, whereas the classical model adequately described the relatively homogeneous aliphatic regions. The fitted CP-dynamic parameters provided effective comparative descriptors of the investigated HAs under the experimental conditions employed and demonstrated consistent depth-dependent differences within the investigated peat profile. These depth-dependent differences may reflect changes associated with peat accumulation and long-term cryogenic preservation; however, this interpretation requires confirmation using independently replicated peat profiles.

## 1. Introduction

The application of advanced high-resolution physicochemical methods has substantially expanded current understanding of the molecular organization of soil organic matter (SOM), its reactivity, and stabilization mechanisms in natural environments. In recent years, particular attention has been devoted to the detailed molecular characterization of SOM using high-resolution spectroscopic techniques, including nuclear magnetic resonance (NMR), Fourier-transform infrared spectroscopy (FTIR), ultrahigh-resolution mass spectrometry, and pyrolytic approaches [1,2,3,4]. The combined use of these analytical methods has significantly improved our understanding of the contribution of aromatic, aliphatic, and carboxyl-containing fragments to the formation of stable soil carbon pools [5,6].

Among SOM components, humic acids (HAs) are traditionally regarded as one of the key fractions controlling the buffering, sorption, and complexation properties of soils [7,8]. Their molecular organization is characterized by pronounced heterogeneity, polydispersity, and structural variability resulting from both the nature of the precursor organic material and the conditions of humification [9,10]. Contemporary concepts no longer interpret HAs as strictly defined macromolecules, but rather as dynamic supramolecular associations of structurally diverse compounds stabilized by non-covalent interactions [11,12]. This structural complexity considerably complicates quantitative characterization and makes the interpretation of analytical data highly sensitive to experimental conditions.

Various chemical fractionation and derivatization procedures, as well as spectroscopic approaches such as UV–Vis and FTIR spectroscopy, often produce inconsistent estimates of aromatic and aliphatic carbon proportions in HAs [13,14,15]. In this context, solid-state ^13^C nuclear magnetic resonance spectroscopy is considered one of the most informative tools for direct characterization of the distribution of carbon functional groups without preliminary chemical modification of the sample. In particular, cross-polarization/magic-angle spinning (CP/MAS) techniques have become widely used for the investigation of both whole soils and insoluble SOM fractions [16,17].

Over the past decade, a substantial number of studies have employed CP/MAS ^13^C NMR spectroscopy to characterize the molecular structure of SOM and HAs from contrasting climatic and ecological settings [18,19,20,21]. Nevertheless, analysis of published datasets reveals considerable methodological variability in spectral acquisition protocols. Reported studies differ substantially in magnetic field strength, MAS spinning frequency, contact time duration, recycle delays, number of scans, proton decoupling schemes, and spectral processing procedures [22,23,24,25].

The quantitative reliability of CP/MAS spectra remains inherently limited because the intensity of ^13^C signals depends not only on the abundance of corresponding carbon functional groups, but also on the efficiency of polarization transfer from ^1^H nuclei and on relaxation characteristics of the system [26,27,28]. In contrast to direct polarization approaches, CP/MAS exhibits unequal sensitivity toward protonated and non-protonated carbon atoms, which may lead to distortion of integral intensities and consequently bias in the interpretation of HA molecular composition [29,30].

It should be emphasized that conventional CP/MAS ^13^C NMR spectroscopy does not provide strictly quantitative carbon distributions because signal intensities are affected by several spin-dynamic factors, including contact time, proton density, molecular mobility, and relaxation processes. Consequently, integrated spectral intensities obtained under CP conditions should be interpreted as apparent signal distributions rather than absolute abundances of carbon functional groups. Nevertheless, when experimental conditions are carefully controlled and identical acquisition parameters are applied, CP/MAS spectra remain highly informative for comparative characterization of molecular organization in complex organic systems.

The efficiency of cross-polarization transfer is strongly governed by spin-dynamic factors, primarily contact time, molecular mobility, proton spin–lattice relaxation time in the rotating frame, and the local spatial arrangement of interacting nuclei. Short contact times preferentially enhance signals from mobile protonated aliphatic structures, whereas longer contact times increasingly favor aromatic and conformationally rigid condensed domains [31,32]. As a consequence, the relative distribution of spectral intensities may reflect not only the intrinsic chemical composition of the sample, but also the dynamics of polarization transfer within different structural environments.

An additional complication arises from relaxation processes in the rotating frame. As previously reported [33,34,35], *T*_1*ρH*_ values can vary across different HA moieties, which may lead to differential signal attenuation during CP experiments [33,34,35]. Variations in relaxation behavior may ultimately alter the apparent contribution of specific molecular fragments to the total spectral intensity distribution. Thus, quantitative interpretation of CP/MAS spectra is intrinsically linked to spin dynamics and cannot be considered independent of experimental acquisition parameters.

Importantly, many published studies provide only limited information regarding acquisition conditions or do not evaluate how these parameters affect calculated structural indices, including aromaticity, hydrophobicity, and O-alkyl carbon contribution. The absence of standardized acquisition protocols complicates direct comparison among datasets and may partly explain inconsistencies reported for chemically similar soil systems.

Although the HA samples investigated in the present study originate from a peat profile that has been previously characterized with respect to elemental composition, conventional CP/MAS ^13^C NMR spectra, and paleoenvironmental interpretation [10], the analytical objectives of the current work are fundamentally different. Earlier research focused primarily on reconstructing environmental changes recorded in peat organic matter, whereas no attempt was made to investigate the spin-dynamic behavior of the samples or to evaluate the influence of experimental parameters on spectral quantification. In the present study, these well-characterized HAs are used as a model system to systematically examine cross-polarization kinetics over a wide range of contact times, to compare classical and extended CP-dynamic models, and to determine effective polarization-transfer and relaxation parameters for individual molecular domains. To our knowledge, such a comprehensive analysis of CP-dynamics in peat HAs has not previously been reported.

Therefore, despite the high analytical value of CP/MAS ^13^C NMR spectroscopy, its application to the apparent structural characterization of HAs remains associated with substantial methodological uncertainty. In particular, the contribution of spin-dynamic factors governing polarization transfer efficiency and signal intensity redistribution has not yet been sufficiently assessed for structurally heterogeneous humic systems.

Accordingly, the aim of the present study was to investigate how spin-dynamic parameters influence CP/MAS ^13^C NMR signal formation in HAs and to determine how polarization transfer kinetics and proton relaxation processes affect the apparent distribution of structural fragments in peat-derived humic systems. Particular attention was paid to evaluating the applicability of classical and extended four-parameter models for describing heterogeneous CP-dynamics and estimating the effective cross-polarization transfer times (*T_CH_*, *T_CH_*_1_, and *T_CH_*_2_) and proton spin–lattice relaxation time in the rotating frame (*T*_1*ρH*_) for different molecular domains of HAs. In addition, the study examined how these CP-dynamic parameters vary among HAs isolated from the seasonally thawed layer (STL), transitional layer (TL), and permafrost layer (PL), and whether they can serve as effective comparative descriptors of differences in CP-dynamic behavior among HAs from different peat horizons under identical experimental conditions.

Beyond the characterization of peat HAs themselves, this work aims to provide methodological recommendations for improving the interpretation of CP/MAS ^13^C NMR spectra of complex natural organic materials and to evaluate the applicability of fitted CP-dynamic parameters as effective comparative descriptors for characterizing differences among samples under the experimental conditions employed.

Because the study was based on a single representative peat profile, the objective was to identify molecular-level relationships within this profile rather than to establish universally applicable mechanisms of HA transformation in permafrost peatlands.

## 2. Results

**Figure 1:**
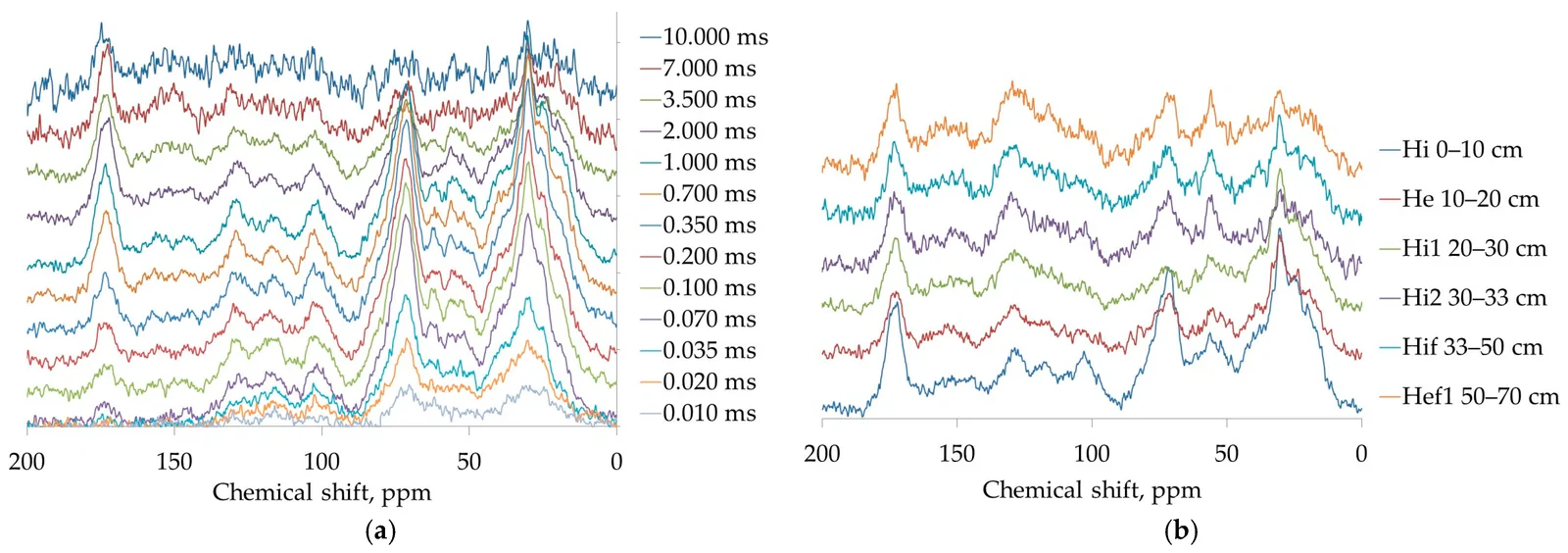
CP/MAS ^13^C NMR spectra: (**a**) HA sample from the Hi layer (with varying contact times); (**b**) HA samples from various horizons of the Hemic Folic Cryic Histosol (with identical contact times of 2.0 ms).

Figure 1 illustrates CP/MAS ^13^C NMR spectra of the HA sample from the upper Hi layer, recorded at various contact times. For completeness, the full set of CP/MAS ^13^C NMR spectra recorded at all contact times for all investigated HA samples is provided in the Supplementary Materials (Figure S1). A visual analysis of the spectra reveals pronounced changes in the overall signal intensity and the ratio of contributions from the different spectral regions as the contact time varies. The most pronounced changes are observed in the C,H-alkyl (0–47 ppm) and O-alkyl (60–108 ppm) regions of the spectra. Increasing the contact time to 0.35 ms causes the signal intensities in these regions to reach their maximum values. Further increases in contact time are accompanied by a gradual decrease in the relative intensity of these signals.

Signals from the carboxyl and amide groups (164–183 ppm) become clearly detectable at contact times exceeding 0.1 ms. By contrast, in the region of aromatic fragments (108–164 ppm), a gradual increase in the relative contribution of signals is observed as contact time increases. Overall, changes in the cross-polarization parameters lead to a noticeable redistribution of intensities between the main structural regions of the spectrum.

**Figure 2:**
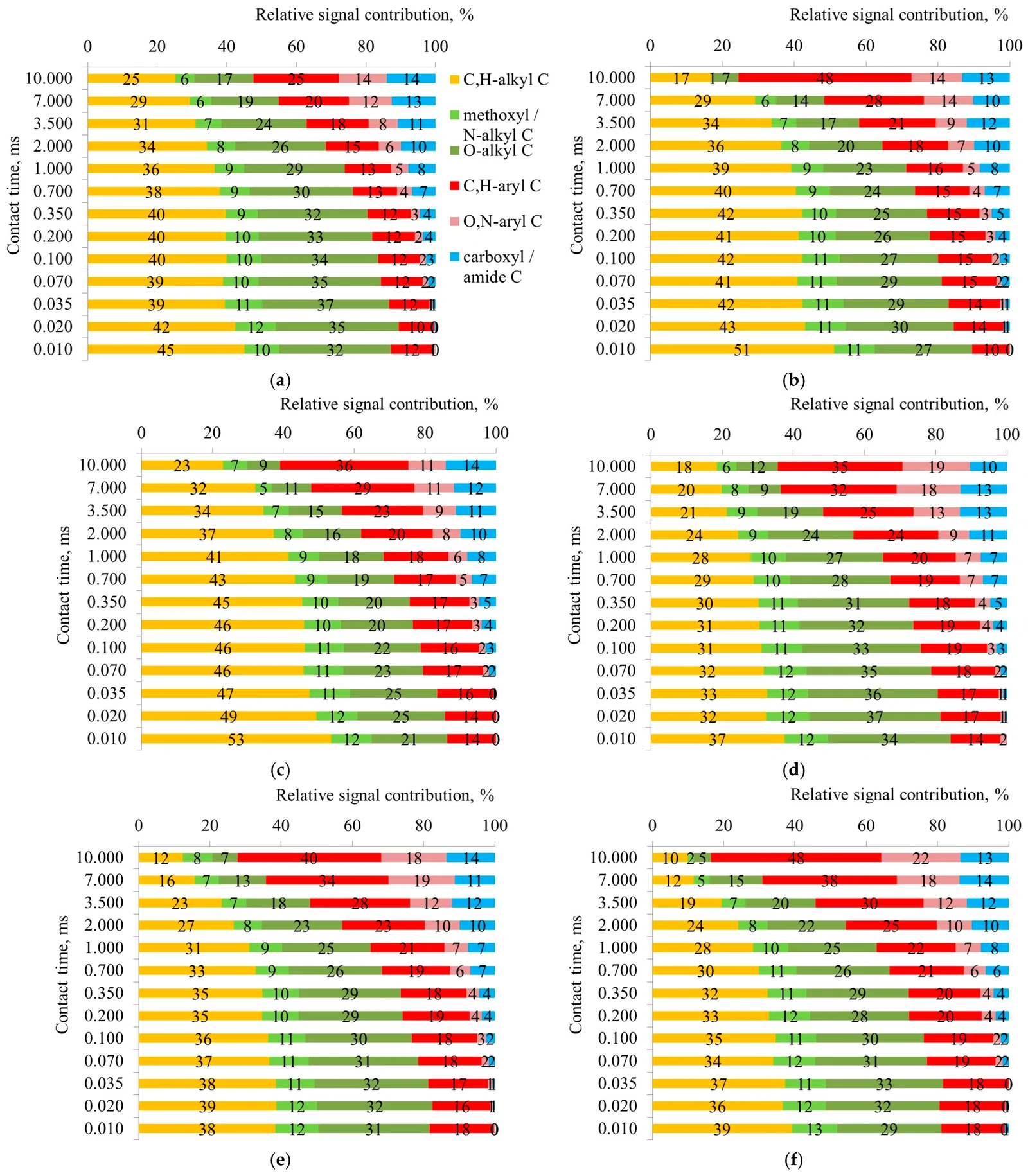
Relative CP/MAS signal distribution among the major molecular fragments of the HA samples based on the CP/MAS ^13^C NMR spectra acquired at different contact times from peat horizons: (**a**) Hi; (**b**) He; (**c**) Hi1; (**d**) Hi2; (**e**) Hif; and (**f**) Hef1.

The results of spectral integration, which characterize the apparent CP/MAS signal distribution among the major functional groups and molecular fragments of the HAs (Figure 2), demonstrate a systematic change in their relative contribution as a function of contact time.

As the contact time increases, the most pronounced decrease is observed for aliphatic and O-alkyl structures, which decrease by several percentage points in relative contribution. At the same time, the contribution of aromatic components increases. The proportion of carboxyl/amide groups shows a particularly noticeable increase, predominantly at longer contact times, indicating an increase in their contribution to the total spectral integral.

**Figure 3:**
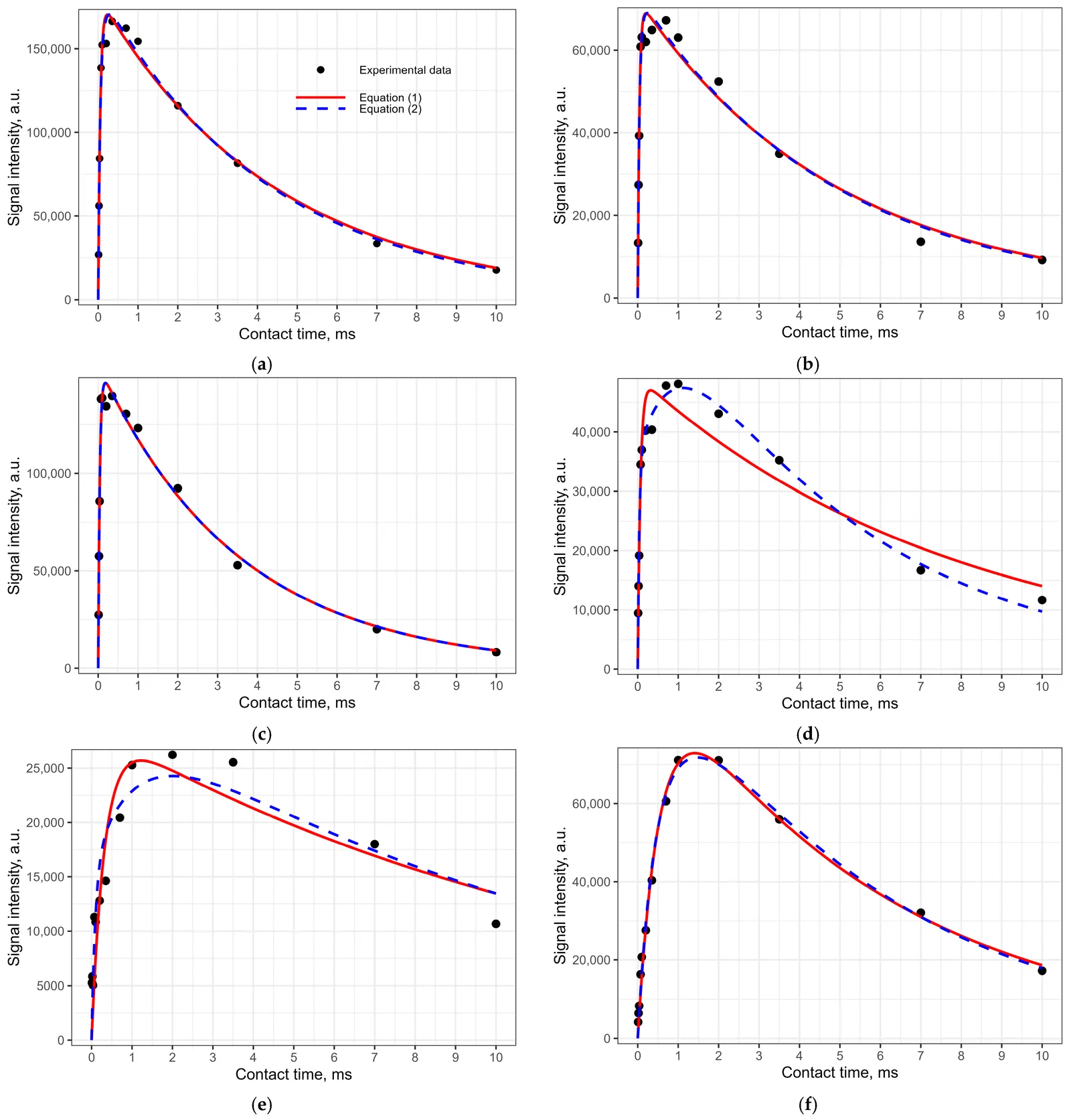
Experimental CP/MAS ^13^C NMR signal intensities and fitted curves of the classical three-parameter and extended four-parameter models as a function of contact time for the HA sample from the Hi horizon: (**a**) C,H-alkyl C; (**b**) methoxyl/N-alkyl C; (**c**) O-alkyl C; (**d**) aryl C; (**e**) O,N-aryl C; (**f**) carboxyl/amide C.

Figure 3 illustrates how the signal intensity of the primary molecular fragments of the HAs varies with contact time. The complete numerical datasets used for nonlinear regression are provided in the Supplementary Materials (Table S1) to facilitate independent evaluation of the fitting procedure.

For all investigated structural groups, the experimental signal intensities showed consistent contact-time-dependent trends that were adequately described by the fitted kinetic models. The different shapes and slopes of the kinetic curves are consistent with differences in cross-polarization behavior and relaxation characteristics among the investigated structural domains of HAs. All of the studied groups exhibited an initial phase of intensity growth at short contact times (*t* < 0.35 ms), followed by gradual signal decay. This indicates the exponential nature of signal relaxation decay and is consistent with theoretical models of CP transfer dynamics in heterogeneous organic systems [31,33]. The complete set of fitted curves for all investigated HA samples is presented in Figure S2.

An analysis of the dependence of CP/MAS ^13^C NMR signal intensities on contact time revealed substantial variation in cross-polarization parameters among functional groups of HAs and among HAs from individual layers of the studied peat profile (Table 1). The experimental data were approximated using two approaches: a classical model (Equation (1)) and an extended four-parameter model (Equation (2)) that accounts for two polarization transfer components. The obtained *T_CH_* and *T*_1*ρH*_ parameters are effective characteristics of CP-dynamics that reflect polarization transfer and relaxation processes in the various structural domains of HAs [17,36].

Overall, the kinetic parameters were estimated with satisfactory precision for most molecular fragments. Larger relative uncertainties were observed primarily for several *T_CH_*_2_ estimates in the O,N-aryl and carboxyl/amide regions, indicating reduced parameter identifiability for these datasets. Accordingly, these parameters are interpreted cautiously and are used only as approximate descriptors of CP-dynamics rather than as precise physical constants.

For all the structural fragments studied, the cross-polarization times were significantly lower than the corresponding *T*_1*ρH*_ values. The ratio *T_CH_* << *T*_1*ρH*_ remained consistent regardless of the nature of the functional group or the depth of the sample. This corresponds to the typical kinetic dependencies of CP/MAS ^13^C NMR and indicates a significantly higher polarization transfer rate than the relaxation attenuation rate of magnetization in a rotating coordinate system. The *T*_1*ρH*_ values varied widely, from 1.87 to 13 ms. Minimum values were primarily characteristic of O-alkyl fragments, while the highest values were observed for the O,N-aryl structures of HAs isolated from STL.

Using the classical model yielded the shortest polarization transfer times for C,H- and O-alkyl groups of the HAs. *T_CH_* values were as follows: C,H-alkyl C: 0.035–0.053 ms; methoxyl/N-alkyl C: 0.035–0.047 ms and O-alkyl C: 0.030–0.041 ms. *T_CH_* values for C,H-aryl fragments increased to 0.036–0.066 ms. The highest *T_CH_* values were characteristic of the O,N-aryl and the carboxyl/amide groups, reaching 0.15–0.59 ms. The maximum value of 0.59 ± 0.07 ms was observed for the carboxyl/amide groups of the HAs from the Hi layer.

For aliphatic HA structures, there was a trend of decreasing *T_CH_* values down the profile. For example, the *T_CH_* parameter for alkyl C decreased from 0.053 ± 0.004 ms in HAs from the upper Hi layer to 0.035–0.036 ms in HAs from the lower Hi and Hef1 layers. A similar decrease was observed for methoxyl/N-alkyl fragments. *T_CH_* values for O-alkyl C remained relatively stable along the profile, varying within the range of 0.030–0.034 ms.

*T*_1*ρH*_ values for aliphatic groups in the HAs decreased with depth along the profile. The *T*_1*ρH*_ parameter for C,H-alkyl C varied from 4.4 ± 0.3 ms in HAs from the upper layer to 2.45 ± 0.16 ms in HAs from the Hef1 layer. The minimum *T*_1*ρH*_ value for O-alkyl C was recorded in the Hi2 layer at 1.87 ± 0.23 ms. Meanwhile, the C,H- and O,N-aryl fragments of the HAs were characterized by longer relaxation times. *T*_1*ρH*_ values for C,H-aryl were predominantly in the range of 5.0–8.0 ms, while *T*_1*ρH*_ values for O,N-aryl C reached 10–13 ms.

The classical model provided high-quality approximations for most aliphatic structural fragments of the HAs. The coefficient of determination (*R*^2^) values for C,H-alkyl, methoxyl/N-alkyl, and O-alkyl C were predominantly in the range of 0.973–0.993. The approximation quality for C,H-aryl C was somewhat lower, particularly in the upper layer (Hi), where the *R*^2^ value was 0.929. O,N-aryl fragments showed the poorest model fit, as indicated by the lowest *R*^2^ values (0.799–0.901) and the highest root mean square error (RMSE) values.

The extended four-parameter model (Equation (2)) described the experimental CP build-up curves using two mathematical components characterized by the fitted parameters *T_CH_*_1_ and *T_CH_*_2_. These parameters represent effective descriptors of the faster and slower components of the adopted fitting function and should not be interpreted as direct evidence for physically distinct polarization-transfer channels. For most aliphatic groups, the *T_CH_*_1_ values were similar to the cross-polarization times calculated using the classical model. At the same time, the *T_CH_*_2_ values were higher and more variable.

*T_CH_*_1_ values for alkyl C ranged from 0.034 to 0.047 ms, while *T_CH_*_2_ values ranged from 0.053 to 0.13 ms. Similar relationships were observed for methoxyl/N-alkyl and O-alkyl fragments, for which *T_CH_*_2_ values were predominantly in the range of 0.045–0.10 ms. For C,H-aryl C of HAs from the upper Hi layer, the differences between the two polarization transfer components were particularly pronounced. *T_CH_*_1_ was 0.040 ± 0.006 ms, whereas *T_CH_*_2_ increased to 1.6 ± 0.6 ms. *T_CH_*_2_ values of C,H-aryl C of HAs from the remaining layers were significantly lower, ranging from 0.053 to 0.082 ms.

The highest apparent *T_CH_*_2_ values were obtained for the O,N-aryl and carboxyl/amide regions. For the O,N-aryl groups, *T_CH_*_2_ ranged from 1.3 to 2.7 ms, whereas values of 1.6–2.4 ms were obtained for the carboxyl/amide region. However, for several O,N-aryl and carboxyl/amide signals, the uncertainty of the fitted *T_CH_*_2_ parameter was comparable to the parameter estimate itself, indicating limited parameter identifiability and reduced robustness of the nonlinear fit. Consequently, these *T_CH_*_2_ values should be regarded as approximate effective kinetic descriptors rather than as accurately determined physical parameters, and differences among individual samples should therefore be interpreted with appropriate caution.

The extended four-parameter model improved the statistical fit for selected structurally heterogeneous spectral regions, with the most pronounced improvement observed for C,H-aryl and O,N-aryl C. In contrast, the fit statistics for the aliphatic regions changed little between the two models.

Despite the improved overall statistical performance of the extended model, the precision of individual fitted parameters differed among spectral regions. In particular, estimates of *T_CH_*_2_ for several O,N-aryl and carboxyl/amide signals exhibited relatively large uncertainties, reflecting the limited amount of information available for resolving multiple polarization-transfer components from the experimental variable-contact-time data. Therefore, the interpretation of these parameters was restricted to qualitative comparison of effective CP behavior rather than detailed mechanistic analysis.

For C,H-aryl fragments of HAs from the Hi layer, the *R*^2^ value increased from 0.929 to 0.985, and the RMSE decreased from 3650 to 1680. For O,N-aryl C, the extended model led to an increase in *R*^2^ and a decrease in RMSE for all HA samples.

For aliphatic HA structures, however, the differences between the two models were less pronounced. The *R*^2^ and RMSE values for C,H-alkyl, methoxyl/N-alkyl, and O-alkyl C remained similar regardless of the approach, indicating that the experimental data were adequately described by the classical three-parameter model.

Overall, the results demonstrate substantial heterogeneity of CP-dynamics among the investigated HA structural fragments. Aliphatic and O-alkyl groups consistently exhibited rapid polarization transfer and relatively short proton spin-lock relaxation times. Aromatic and heteroatom-containing fragments generally displayed slower apparent polarization-transfer behavior. However, because estimates of *T_CH_*_2_ for some O,N-aryl and carboxyl/amide regions were associated with relatively large uncertainties, these values should be regarded primarily as effective comparative descriptors of CP kinetics rather than as definitive evidence for distinct molecular polarization-transfer mechanisms.

## 3. Discussion

The results obtained indicate a pronounced dependence of signal intensities in CP/MAS ^13^C NMR spectra on contact time, one of the cross-polarization method’s fundamental features. The redistribution of signal contributions among the different structural domains of HAs results from magnetization transfer from ^1^H to ^13^C nuclei and is governed by the spin dynamics of the CP process [31,36].

A limitation that should be considered when interpreting the integrated spectral regions is the potential contribution of spinning sidebands. The spectra were acquired at a MAS frequency of 12.5 kHz, which was expected to reduce sideband contributions; however, no sideband-suppression experiment or measurements at a second MAS frequency were performed. Consequently, the extent to which sidebands contributed to the defined integration windows cannot be quantified from the present dataset. This limitation is particularly relevant because some of the observed changes in relative spectral contributions were on the order of several percentage points. Although the systematic contact-time dependence of the spectra is consistent with changes in CP transfer and relaxation, the present data do not allow the sideband contribution to be completely separated from the intrinsic CP-dependent changes in integrated signal intensity. Therefore, the integrated areas are best interpreted as apparent CP/MAS signal distributions under the applied experimental conditions rather than as strictly quantitative measures of the abundance of individual carbon groups.

The increase in signal intensity of C,H- and O-alkyl fragments at short contact times likely results from the high proton density and effective dipole–dipole interactions in these structural elements. For proton-saturated fragments, polarization transfer occurs most efficiently, leading to rapid attainment of maximum signal intensity. Similar patterns have been described previously for various types of SOM [17,35,37].

In contrast, the slower increase in signal intensity of aromatic and carboxyl structures of the HAs is attributed to the smaller number or absence of directly bound protons. Consequently, the efficiency of CP transfer decreases, and the maximum signal intensities shift toward longer contact times. This behavior is consistent with the current understanding of cross-polarization mechanisms in chemically and structurally heterogeneous organic systems [33,38].

The calculated *T*_1*ρH*_ values differ considerably among the functional groups of the HAs. The lower values characteristic of C,H- and O-alkyl structures are consistent with greater molecular mobility of these fragments; however, relaxation behavior is influenced by multiple experimental and structural factors, and this interpretation should therefore be regarded as tentative. Conversely, the elevated *T*_1*ρH*_ values for aromatic structures indicate a more condensed and structured state, consistent with existing concepts regarding HA organization [39,40].

Of particular interest is the high *T*_1*ρH*_ value for O,N-aryl structures. This is likely due to stable intermolecular interactions, such as hydrogen bonds and π–π associations between aromatic fragments, that restrict local molecular mobility [41,42]. However, interpreting this effect requires caution since relaxation characteristics may simultaneously be influenced by several factors, including the heterogeneity of the local carbon atom environment and the distribution of molecular motion correlation times.

Overall, the results indicate that using CP/MAS ^13^C NMR spectroscopy to assess the apparent molecular organization of HAs without considering cross-polarization kinetics and relaxation processes may result in substantial changes in the apparent integral ratios of spectral regions. Comparative analysis of different natural polymers, including HAs, is meaningful only when the samples have sufficiently similar molecular compositions. This issue has been discussed repeatedly in the literature, which emphasizes the need to standardize CP/MAS experimental parameters in comparative studies of SOM in general and HAs in particular [43,44,45].

These observations further emphasize that conventional single-contact CP/MAS experiments should not be interpreted as providing absolute molecular composition. Rather, the obtained spectral integrals represent apparent signal distributions that reflect both the chemical composition of HAs and the efficiency of polarization transfer within individual structural domains. Consequently, direct comparison of absolute integral values among studies employing different acquisition parameters should be approached with caution.

Additionally, the varying shapes of the kinetic dependencies of individual structural groups suggest distributions of cross-polarization and relaxation times. These distributions reflect the high chemical and structural heterogeneity of HAs. Each spectral region likely includes a set of fragments with different local environments and polarization transfer efficiencies, corresponding to the concept of HA complex supramolecular organization [38,46].

One of the observations made under the applied experimental conditions is a consistent decrease in the absolute CP/MAS signal intensity with depth within the studied peat profile (Figure 3). However, because no intensity normalization to dry mass, carbon content, or an external standard was performed, and complete proton magnetization recovery was not verified, this depth-related variation must be regarded as a comparative observation only. Since all samples were analyzed using identical acquisition parameters, receiver gain, rotor filling protocol, packing conditions, probe tuning, and the same number of scans, experimental variability associated with instrumental settings was minimized. Nevertheless, because no external intensity standard or quantitative normalization procedure was employed, instrumental and sample-preparation effects cannot be excluded completely. Therefore, the observed decrease in absolute CP/MAS signal intensity with depth should be regarded as a comparative observation rather than direct quantitative evidence. It is possible that changes in cross-polarization efficiency contribute to the observed trend, but without normalization to sample mass or carbon content, or to an external intensity standard, and without verification of complete proton magnetization recovery, no mechanistic interpretation can be assigned. The efficiency of CP transfer is known to be determined not only by carbon content, but also by proton density, the spatial proximity of ^1^H and ^13^C nuclei, and the molecular mobility of the organic matrix [36,47]. Consequently, differences in cross-polarization efficiency arising from changes in molecular organization appear to provide the most reasonable explanation for the observed trend, although additional experiments employing quantitative reference methods would be required for unambiguous verification.

Another factor may be the age of the SOM. The PL studied formed more than 3600 years ago [10] and consists of plant material from the pre-industrial period. Theoretically, this could lead to slight differences in the natural ^13^C isotope content compared with that of modern plants. However, known variations in the natural isotopic distribution of ^13^C in terrestrial vegetation are relatively small and unlikely to explain the multiple reductions in signal intensity observed. Thus, while differences in cross-polarization efficiency and HA molecular organization may play a role, the current data do not permit a definitive causal interpretation.

The observed depth-related variations are consistent with the current understanding of SOM transformation in permafrost peat deposits. Previous studies have shown that, during peat development, the relative contribution of readily degradable aliphatic components generally decreases, whereas aromatic and oxygen-containing structures become relatively more abundant [5,48]. Under prolonged cryogenic conditions these transformations are believed to proceed more slowly, potentially contributing to increased structural heterogeneity and more complex supramolecular organization of SOM [11,38]. The present results are compatible with this interpretation; however, because only a single peat profile was investigated, they should be regarded as supporting rather than independently demonstrating these processes.

The behavior of CP-dynamics parameters in the TL (Hi2) and the underlying PL (Hif and Hef1) warrants special attention. The most pronounced changes in the kinetic parameters of several structural fragments were observed near the boundary between the seasonally thawed and permafrost layers. This interval may represent a transition zone in which organic matter exhibits characteristics consistent with both ongoing humification in the seasonally thawed layer and prolonged preservation under permafrost conditions. Similar transition zones have previously been described as regions where substantial changes in organic matter composition may occur [10,49,50]. Because the present study was limited to a single peat profile, these observations should be regarded as descriptive and consistent with previous reports rather than as direct evidence for the underlying mechanisms.

A comparison of the parameters calculated using the classical and extended models (Table 1) reveals that their informative value significantly depends on the nature of the structural fragments under study. For C,H-alkyl, methoxyl/N-alkyl, and O-alkyl groups, the models produced similar results. The values of the coefficients of determination remained high regardless of the approximation method, and the *T_CH_* and *T_CH_*_1_ parameters were nearly identical. These results suggest the relative homogeneity of the local environment of aliphatic structures and the presence of a predominant polarization transfer mechanism. These fragments are likely primarily represented by residues of plant biopolymers and products of their partial transformation within HA molecules. These molecules retain a sufficiently high proton density and similar conditions for dipole–dipole interactions.

The goodness-of-fit statistics summarized in Table 2 (*R*^2^, RMSE, AIC, and BIC) provide a more comprehensive assessment of model performance than the coefficient of determination alone. For the aliphatic carbon groups (C,H-alkyl, methoxyl/N-alkyl, and O-alkyl C), the classical monotonic model consistently yielded lower AIC and BIC values than the extended model, indicating that the additional parameters did not improve the description of the experimental CP build-up curves sufficiently to justify the increased model complexity. In contrast, the extended model showed clear advantages for several structurally more heterogeneous carbon environments. The largest improvement was observed for aromatic carbon in the upper peat layer (Hi), where the decrease in AIC exceeded 18 units, providing strong evidence in favor of the extended model [51]. For O,N-aryl carbon, lower AIC and BIC values were obtained for most peat layers, indicating that multicomponent CP kinetics more adequately describe these chemically heterogeneous structural domains. A similar tendency was observed for carboxyl/amide carbon in all layers except the uppermost horizon. These results demonstrate that the applicability of the extended model depends on the molecular environment and that increased model complexity is justified primarily for structurally heterogeneous functional groups, whereas the classical model adequately describes the CP-dynamics of relatively homogeneous aliphatic fragments.

A different picture emerged for aromatic and carboxylic structures. For these groups, applying the four-parameter model notably improved the quality of the approximation, particularly for samples from the STL. The improved fit obtained with the four-parameter model indicates that the experimental CP build-up curves are more adequately described by a function containing fast and slow mathematical components. Although these components may be consistent with differences in local molecular environments, the present data do not allow assignment of *T_CH_*_1_ and *T_CH_*_2_ to specific polarization-transfer mechanisms or particular structural domains. This effect is most pronounced for O,N-aryl and carboxylic fragments, which have high *T*_1*ρH*_ and *T_CH_*_2_ values simultaneously.

It should be noted that the highest parameter estimation errors and lowest values of the coefficients of determination were specifically observed for O,N-aryl structures. This may indicate the high chemical heterogeneity of this spectral region rather than a shortcoming of the model used. In the 144–164 ppm range, signals from phenolic, lignin-like, and quinoid structures are recorded simultaneously. These structures can differ significantly in molecular mobility and CP-transfer efficiency [6,17,52]. Consequently, the observed kinetic dependence is a superposition of several processes, each with its own cross-polarization and relaxation times.

For most aliphatic structures, the effective *T_CH_* and *T*_1*ρH*_ values generally decreased from the upper STL toward the deeper PL. This trend is consistent with progressive changes in the molecular organization of HAs during peat development. However, because the present study examined only one representative HA sample from each horizon, these observations should be interpreted as descriptive trends within the investigated peat profile rather than statistically validated evidence of diagenetic transformation. The relatively high *T*_1*ρH*_ values observed for aromatic structures throughout the profile may indicate the persistence of comparatively rigid structural domains, although confirmation of this interpretation will require analyses of additional peat profiles. Previous studies of peat of various genesis and degrees of transformation have noted a similar combination of shortened relaxation times for aliphatic components and relative stability of aromatic structures [9,29].

From a broader analytical perspective, the present study demonstrates that CP/MAS ^13^C NMR spectra of HAs cannot be interpreted quantitatively without considering spin-dynamic effects associated with polarization transfer and relaxation processes. The results show that contact time substantially modifies the apparent distribution of carbon functional groups and that the extended CP-dynamic model can improve the description of selected structurally heterogeneous spectral regions, whereas the classical model remains sufficient for relatively homogeneous aliphatic domains. Unlike previous studies of these peat samples, which were primarily focused on environmental reconstruction, the present work provides new methodological insight into the quantitative interpretation of solid-state NMR data. The obtained CP-dynamic parameters may therefore be regarded as additional molecular descriptors reflecting supramolecular organization and structural heterogeneity of natural organic matter. These findings expand the analytical capabilities of solid-state NMR spectroscopy and are relevant not only for soil science, but also for molecular characterization of a wide range of heterogeneous organic materials.

It should also be emphasized that the extended model provides an effective phenomenological description of CP build-up kinetics rather than a unique microscopic description of polarization-transfer processes. Although the additional parameter *T_CH_*_2_ improves the mathematical representation of heterogeneous CP behavior, its uncertainty was relatively large for several O,N-aryl and carboxyl/amide regions. Accordingly, these estimates should be interpreted primarily in a comparative sense and should not be regarded as direct evidence for specific molecular-scale polarization-transfer mechanisms.

Because only one representative HA sample from each peat layer was investigated, the observed depth-related variations describe the behavior of the studied peat profile and should not be interpreted as statistically validated differences among independent peat populations. The regression-derived uncertainties presented in this work quantify the precision of the fitted CP kinetic parameters rather than natural spatial variability within peat deposits. Verification of the generality of the observed trends will require future studies including independent field replicates from multiple peat profiles.

## 4. Materials and Methods

### 4.1. Study Site and Sampling

HA samples were isolated from six horizons: Hi, He, Hi1 (STL), Hi2 (TL) and Hif, Hef1 (PL) of a permafrost peat soil classified as a Hemic Folic Cryic Histosol [53]. The soil profile included both STL and the underlying PL. The STL was sampled from open pits at 10 cm depth intervals. The PL was sampled using a steel corer at 20 cm intervals, and intact frozen core sections were recovered between drilling points.

One representative HA sample was isolated from each investigated peat layer and analyzed by variable-contact-time CP/MAS ^13^C NMR. Thus, the present study compares six representative HA samples originating from different depths of a single peat profile rather than independent biological replicates. Consequently, the observed depth-related differences should be interpreted as descriptive trends within the investigated profile and not as statistically demonstrated population-level differences among peat layers.

**Figure 4:**
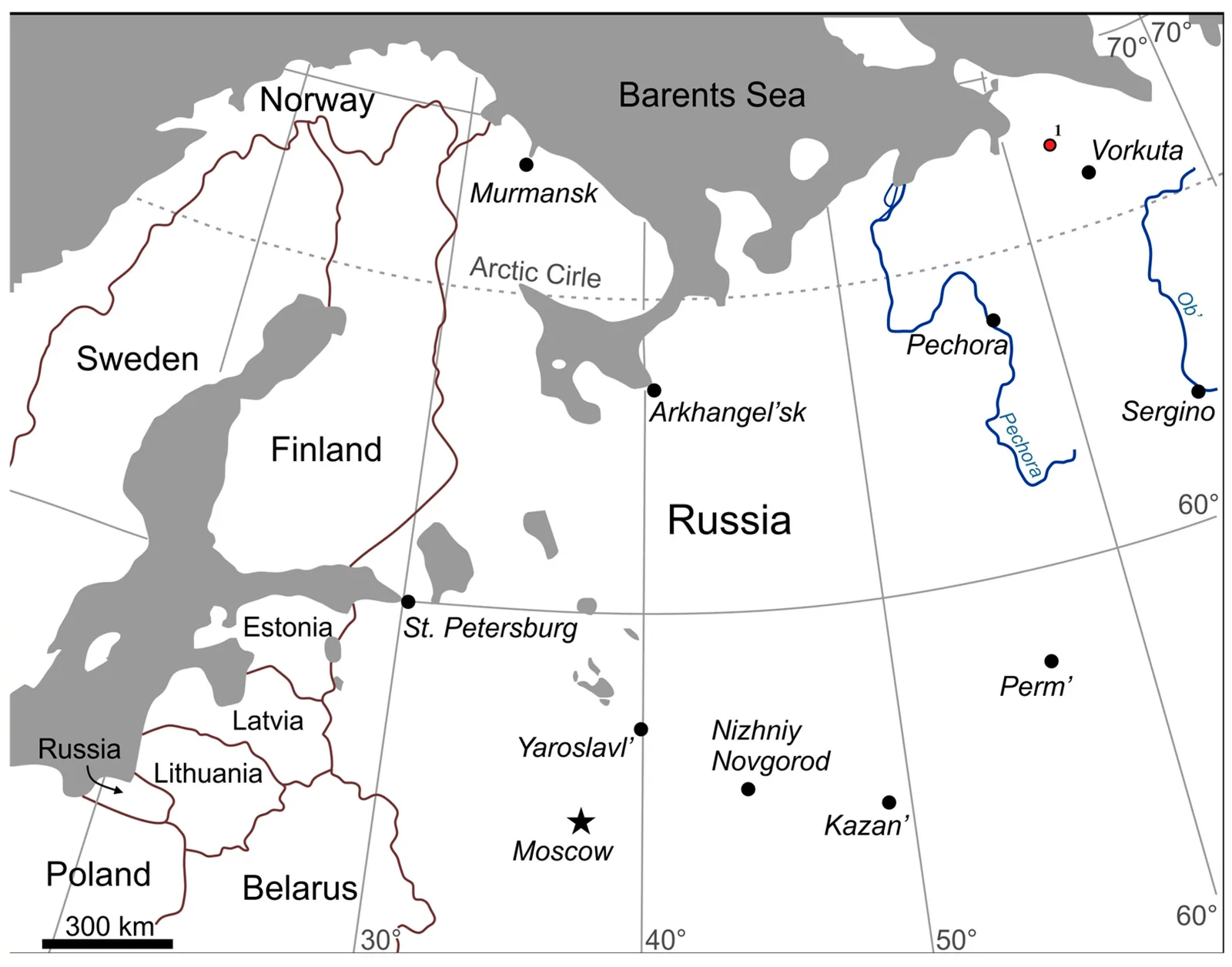
Location of sampling point (1).

The investigated peatland (Figure 4) is situated in the ecotone between the north and south tundra (Korotaikha River basin, Nenets Autonomous Okrug, Russia; 68°2′09″ N, 62°43′45″ E). The study plot occupies a flat, waterlogged lacustrine-alluvial valley (30–100 m a.s.l.) characterized by low topographic relief and extensive wetland massifs. Permafrost is widespread in this region [54], with the upper boundary occurring at a depth of 33 cm.

The investigated site exhibits well-defined microrelief primarily shaped by cryogenic processes. Approximately 60% of the massif consists of hummocks (0.8–1.5 m high), while the remaining area comprises swampy hollows, swales, inundated depressions, and secondary lakes. Vegetation cover is characterized by polydominant fruticose, moss, and lichen communities. The ground cover is primarily composed of green mosses (e.g., *Polytrichum strictum*, *Pleurozium schreberi*, *Dicranum elongatum*) and *Sphagnum* species (*S. fuscum* and *S. compactum*), while prevalent lichens include *Flavocetraria nivalis* and *Cladonia* sp. Dominant herbaceous and dwarf shrub species include *Empetrum hermaphroditum*, *Ledum palustre*, *Rubus chamaemorus*, *Vaccinium uliginosum*, and *Betula nana*. Detailed physicochemical properties, peat botanical composition, and morphological descriptions of the studied profile were reported previously [10].

The mass fraction of carbon in the HAs of the studied soil ranges from 56.0 to 58.0%. The nitrogen and hydrogen contents in the samples tend to decrease with depth in the peat profile. The mass fraction of ash in the samples does not exceed 1% (Table 3). Detailed data on the elemental composition of the studied HAs have been published previously [10].

### 4.2. Sample Preparation and HA Separation

HA samples were extracted using the International Humic Substances Society (IHSS) method [55]. The extraction process involved treating 50 g of air-dried peat twice with a 0.1 mol/dm^3^ NaOH solution at a solid-to-liquid ratio of 1:50. The resulting alkaline extract underwent coagulation of colloidal particles by the addition of NaCl, reaching a final concentration of 0.3 mol/dm^3^, after which the extract was centrifuged for one hour at 10,000 rpm using a SIGMA 2-16 KL ultracentrifuge (Sigma Laborzentrifugen GmbH, Osterode am Harz, Germany).

The HAs were precipitated by gradually adding 6 mol/dm^3^ HCl until the pH reached 1.1–1.2. To remove mineral impurities, the preparation was purified using a mixture of HCl and HF solutions at concentrations of 0.1 and 0.3 mol/dm^3^, respectively. Then, the HAs were dialyzed until the low-molecular-weight compounds were removed. The obtained HA samples were dried at 35 °C in a forced-convection oven, ground, and sieved through a 0.1 mm mesh sieve.

The isolated HAs represent the operationally defined alkaline-extractable humic acid fraction of peat organic matter obtained under the applied extraction and purification procedure, rather than the entire native organic matter pool.

### 4.3. CP/MAS ^13^C NMR Spectroscopy

We recorded CP/MAS ^13^C NMR spectra of air-dried HA samples on a Bruker Avance III WB 400 spectrometer (Bruker BioSpin GmbH, Rheinstetten, Germany) operating at 100.6 MHz for ^13^C nuclei at the Scientific Park of St. Petersburg State University. The CP/MAS method with magic-angle sample spinning was used.

Samples were packed into 4 mm zirconia rotors equipped with KelF caps (Wilmad-LabGlass, Vineland, NJ, USA). The mass of air-dried HAs loaded into each rotor was approximately 80 mg. The samples were equilibrated to constant mass in the laboratory atmosphere (relative humidity ~40%). No normalization of the recorded signal intensities to sample mass or an external standard was applied; therefore, all intensities are reported directly in arbitrary units as obtained from the integrated spectra.

Magic-angle spinning was carried out at 12.5 kHz. The temperature was kept constant at 25.0 ± 0.1 °C. The Hartmann–Hahn matching condition was optimized prior to the experimental series and kept constant throughout all measurements. Information about the cross-polarization pulse shape (constant-amplitude or ramped-amplitude) could not be retrieved from the available experimental records; therefore, this detail is not reported. The absence of this specification is acknowledged as a limitation of the experimental description. The ^1^H 90° pulse length was 2.3 μs (58 W), corresponding to a radiofrequency field strength of 110 kHz, while the ^13^C transmitter power setting was 105 W. The corresponding ^13^C nutation frequency (B1 field) was not separately calibrated. CP/MAS ^13^C NMR spectra were acquired using 13 contact times of 0.01, 0.02, 0.035, 0.07, 0.1, 0.2, 0.35, 0.7, 1, 2, 3.5, 7, and 10 ms.

High-power proton decoupling was applied during acquisition using the SPINAL-64 decoupling scheme at a decoupling field strength of 57.6 kHz. The spectral width was 30.2 kHz (300.5 ppm), the acquisition time was 33.9 ms, and 2048 complex data points were collected. A recycle delay of 2 s was employed, and each spectrum was acquired using 512 scans. The receiver gain was set to 203 and kept constant throughout the experiments. Probe tuning and matching were performed prior to each measurement.

A recycle delay of 2 s was selected as a compromise between the experimental duration and recovery of proton magnetization and was kept identical for all measurements. Proton spin–lattice relaxation times (^1^H T1) were not measured for the investigated HA samples. Consequently, complete recovery of proton magnetization between successive scans cannot be verified for every sample. The experimental protocol was therefore designed for comparative analysis under identical acquisition conditions rather than for absolute quantitative measurements.

Chemical shifts were referenced to tetramethylsilane (TMS, 0 ppm) as an external reference.

All spectral processing was performed using MestReNova^®^ v.14.2.0 (Mestrelab Research S.L., A Coruña, Spain). The free induction decays were multiplied by an exponential line-broadening function equivalent to 50 Hz (Lorentzian broadening) prior to Fourier transformation. No additional zero-filling was applied beyond the acquired 2048 complex data points. Phase correction and baseline correction were carried out manually. After these corrections, the spectral regions were integrated with the software’s standard integration tool, without further baseline adjustment. Although the MAS frequency of 12.5 kHz was expected to reduce spinning-sideband contributions, this was not experimentally verified by a sideband-suppression procedure or by repeating the measurements at a different MAS frequency. Therefore, possible contributions of spinning sidebands to the defined integration windows cannot be quantitatively excluded. In particular, because the relative spectral contributions changed by several percentage points with contact time, even a limited and contact-time-dependent sideband contribution could affect the integrated areas. The resulting spectral integrals should therefore be interpreted as apparent CP/MAS signal distributions rather than strictly quantitative estimates of carbon-group abundances.

For kinetic modelling, the integrated intensities measured at different contact times were exported and fitted using nonlinear regression procedures described below. The complete numerical integration dataset together with the fitted signal intensities used for kinetic modelling is provided in the Supplementary Materials (Table S1).

Because CP/MAS signal intensities depend on cross-polarization efficiency, proton density, molecular mobility, and relaxation behavior, the obtained values are discussed as apparent CP/MAS signal distributions rather than absolute quantitative carbon abundances [27]. To characterize the relative distribution of carbon functional groups, the spectra were integrated over the following chemical shift ranges:

0–47 ppm (C,H-alkyl C): aliphatic C,H-substituted fragments of polymethylene and branched paraffin structures;

47–60 ppm (methoxyl/N-alkyl C): methoxyl groups and N-substituted aliphatic fragments;

60–108 ppm (O-alkyl C): carbohydrates and other O-substituted aliphatic structures;

108–144 ppm (C,H-aryl C): C,H-substituted aromatic fragments;

144–164 ppm (O,N-aryl C): O- and N-substituted aromatic structures, predominantly phenolic;

164–183 ppm (carboxyl/amide C): carboxyl, amide, and ester groups.

This integration scheme is consistent with the standard approach for interpreting CP/MAS ^13^C NMR spectra of SOM and HAs [17,56]. These chemical-shift regions represent operational integration windows commonly used for the interpretation of CP/MAS ^13^C NMR spectra of SOM and HAs [35]. Because several regions contain overlapping resonances from chemically distinct carbon environments, the assigned structural groups should be regarded as approximate rather than exclusive.

### 4.4. Evaluation of Cross-Polarization Parameters

The signal intensity in CP/MAS ^13^C NMR spectra depends on the efficiency of polarization transfer from ^1^H nuclei to ^13^C nuclei, as well as on proton relaxation behavior, molecular mobility, dipolar interactions, and the local structural environment. In single-contact experiments, the primary parameters affecting signal formation are *T_CH_*, which determines the rate of magnetization transfer, and *T*_1*ρH*_, which influences the decay rate of proton magnetization in the spin-lock regime.

The dependence of CP/MAS signal intensity on contact time was analyzed using the classical phenomenological CP kinetic model, which has been widely applied for the interpretation of variable-contact-time CP/MAS experiments because it provides effective parameters describing cross-polarization efficiency and proton spin-lock relaxation. Although this model does not represent a complete microscopic description of CP spin dynamics, it remains one of the most commonly used approaches for comparative characterization of structurally heterogeneous organic materials [17]. The dependence of signal intensity on contact time was described by the following equation:

*I_t_* = *I*_0_ (1 − exp(−*t*/*T_CH_*)) exp(−*t*/*T*_1_*_ρH_*), (1)

where *I*_0_ represents the apparent maximum CP/MAS signal intensity attainable under the applied experimental conditions and is proportional to the number of observable ^13^C nuclei contributing to the CP experiment. Accordingly, *I*_0_ should be regarded as an effective CP parameter rather than an absolute quantitative measure of carbon abundance.

This model assumes a single effective polarization-transfer process and describes a single CP build-up component followed by proton spin-lock relaxation. However, for structurally heterogeneous systems such as HAs, differences in molecular mobility, proton density, and the distribution of local molecular environments may result in deviations from simple monotonic CP kinetics.

To provide a more flexible description of CP kinetics in structurally heterogeneous HAs, an extended four-parameter semi-empirical model was employed:

*I_t_* = *I*_0_ (1 − 0.5exp(−*t*/*T_CH_*_1_) − 0.5exp(−3*t*/2*T_CH_*_2_)) exp(−*t*/*T*_1_*_ρH_*), (2)

where *T_CH_*_1_ and *T_CH_*_2_ represent the effective fast and slow mathematical components of the adopted phenomenological fitting function. Although these components may reflect differences in CP behavior arising from heterogeneous molecular environments, they should not be interpreted as experimentally demonstrated assignments to specific structural domains or distinct polarization-transfer mechanisms. A multicomponent phenomenological approach for describing CP kinetics in heterogeneous natural organic matter was proposed by Conte and Berns [33] as an extension of earlier phenomenological descriptions of CP-dynamics [17]. The equation employed in the present study follows the same phenomenological concept but represents a simplified formulation intended for comparative analysis of the investigated HAs. Unlike the original model of Conte and Berns [33], the equation used here does not include the dipolar coupling term describing heteronuclear dipolar interactions. Consequently, the adopted equation contains fewer adjustable parameters and provides a robust mathematical description of the experimental variable-contact-time CP/MAS data while retaining sufficient flexibility to account for the structural heterogeneity of the investigated HAs. Accordingly, it should be regarded as a simplified phenomenological approximation rather than as a rigorous microscopic description of CP spin dynamics.

The parameters *T_CH_*_1_ and *T_CH_*_2_ should therefore be interpreted as effective kinetic descriptors corresponding to apparent fast and slow polarization-transfer components rather than as independent physical relaxation constants. Likewise, the weighting coefficients and exponential terms are elements of the adopted fitting function introduced to improve the description of the experimental CP build-up curves and should not be interpreted as possessing independent microscopic physical significance. The model was employed to obtain effective kinetic descriptors for comparative characterization of HAs under identical experimental conditions rather than to establish a unique physical description of the underlying spin-dynamic processes.

Nonlinear regression was performed in the R statistical environment (version 4.5.3, RStudio IDE, Posit PBC, Boston, MA, USA) using the Levenberg–Marquardt optimization algorithm implemented in the *minpack.lm* package. Parameter bounds were introduced to ensure physically meaningful solutions and to improve numerical convergence. For both models, all fitted parameters were constrained to positive values; additionally, in the extended four-parameter model the characteristic polarization-transfer times *T_CH_*_1_ and *T_CH_*_2_ were restricted to the ranges 0.001–0.5 ms and 0.001–10 ms, respectively. The complete R scripts used for nonlinear regression, including the input data, model definitions, Levenberg–Marquardt fitting, bootstrap procedures, and calculation of AIC and BIC, are provided as a Supplementary Code S1 (file “CP_kinetics_fitting.R”).

The quality of model fitting was evaluated using the coefficient of determination (*R*^2^), root mean square error (RMSE), Akaike Information Criterion (AIC), and Bayesian Information Criterion (BIC). The latter two criteria were used to compare the goodness of fit of the classical and extended models while accounting for differences in model complexity. AIC and BIC were calculated according to the standard formulas:

AIC = *n*∙ln(RSS/*n*) + 2*k* (3)

BIC = *n*∙ln(RSS/*n*) + *k*∙ln(*n*), (4)

where *n* = 13 (number of contact-time points per kinetic curve), *k* = 3 for the classical model and *k* = 4 for the extended model, and RSS is the residual sum of squares. The calculations were performed using the built-in functions AIC() and BIC() of the stats package in R (version 4.5.3, RStudio IDE).

Because the experimental CP/MAS signal intensities were expressed in arbitrary units (a.u.), RMSE values are reported in the same units. Because RMSE depends on the magnitude of the experimental signal intensity, RMSE values were used only to compare alternative models fitted to the same dataset and were not interpreted across different HA samples.

For the classical model, parameter uncertainty was estimated from the covariance matrix obtained during nonlinear regression, and approximate 95% confidence intervals were calculated from the estimated parameter variance. For the extended four-parameter model, parameter uncertainty was evaluated by residual bootstrap resampling (1000 iterations). Standard errors, covariance matrices, and 95% confidence intervals were estimated from the bootstrap parameter distributions. Bootstrap analysis was additionally used to assess the stability of the fitted parameters and their practical identifiability. Bootstrap distributions of the parameters are shown in Figure S3. For all fitted models, parameter estimates converged consistently within the specified parameter bounds. Sensitivity analysis with random initial parameter values confirmed that the fitted solutions were insensitive to starting values, with coefficients of variation below 0.01% for the tested datasets (Code S1). For the classical model, the covariance matrices were well-conditioned and confidence intervals remained finite for all fitted parameters. For the extended model, bootstrap resampling indicated that *T_CH_*_1_ and *T*_1*ρH*_ were generally well-determined, whereas *T_CH_*_2_ for several O,N-aryl and carboxyl/amide groups showed wide distributions (relative uncertainty ≥100%; see Table 1). Therefore, while the main parameters are practically identifiable, the *T_CH_*_2_ values marked as poorly constrained should be interpreted with particular caution. Parameter correlation matrices are provided in Figure S4.

The standard errors and confidence intervals reported throughout this study originate from nonlinear regression and bootstrap analysis of the CP kinetic models. They characterize the uncertainty of the fitted kinetic parameters under the applied model and experimental conditions and should not be interpreted as estimates of biological or sampling variability among independent HA samples.

Residuals were examined after nonlinear regression to verify the absence of systematic deviations between experimental and fitted values. In all cases, the residuals were randomly distributed around zero without pronounced systematic trends, indicating that both models adequately described the experimental CP build-up curves. Residual plots for all fits are provided in Figure S5. Model selection was based on the combined evaluation of *R*^2^, RMSE, AIC, BIC, residual analysis, and parameter stability rather than on any single goodness-of-fit statistic.

The obtained parameters *T_CH_*, *T_CH_*_1_, *T_CH_*_2_, and *T*_1*ρH*_ were therefore considered effective descriptors of CP-dynamics reflecting the combined influence of molecular mobility, local proton environment, dipolar interactions, and structural heterogeneity of the HAs. Because CP kinetics are influenced by multiple experimental and molecular factors, these parameters should be interpreted comparatively rather than as unique microscopic physical constants or absolute molecular characteristics.

## 5. Conclusions

A detailed assessment was conducted on the effect of contact time on CP/MAS ^13^C NMR spectra of HAs isolated from permafrost peat soil. The results demonstrated that contact time significantly affects the apparent distribution of CP/MAS signal intensities within each sample and the relative contributions of the major structural domains. Comparisons of absolute signal intensity among independently prepared samples should be regarded only as comparative observations because no intensity normalization was performed. Short contact times preferentially enhance proton-rich aliphatic and carbohydrate structures, whereas longer contact times increase the relative contribution of aromatic and carboxyl-containing fragments. These findings indicate that conventional CP/MAS spectra should not be interpreted as strictly quantitative representations of carbon functional group abundances without considering spin-dynamic effects and other spectral contributions, including potential spinning-sideband contributions.

The kinetics of intensity change for all structural groups investigated is characterized by a rapid increase in signal during the initial stages of cross-polarization, reaching a maximum and subsequently relaxing and decaying. Calculated values *T*_1*ρH*_ showed that minimum relaxation times are characteristic of aliphatic and carbohydrate HA fragments, while maximum values are observed for aromatic and carboxyl structures. The obtained CP-dynamic parameters are consistent with differences in molecular mobility and local proton environments among the investigated HA structural fragments, although these relationships should be regarded as indirect inferences rather than direct measurements.

A comparison of the classical three-parameter and extended four-parameter models of CP-dynamics revealed that their effectiveness depends on the nature of the structural fragments studied. The classical model adequately described CP-dynamics of aliphatic fragments, whereas aromatic and heteroatom-containing structures were more accurately represented by the four-parameter model. The latter provided a more flexible phenomenological description of the experimentally observed CP kinetics in structurally heterogeneous HAs. However, the additional kinetic parameters should be interpreted as effective comparative descriptors of CP behavior rather than as uniquely determined physical characteristics of individual molecular domains. This conclusion is further supported by the comparison of AIC and BIC values, which demonstrated that the extended model substantially improved the description of CP kinetics for aromatic and heteroatom-containing fragments, whereas both models performed similarly for the more homogeneous aliphatic HA structures.

This study’s practical significance lies in substantiating approaches to selecting CP/MAS ^13^C NMR experimental parameters for studying HAs and other complex systems. For comparative studies, it is recommended that researchers use standardized spectral recording conditions and consider the possible influence of CP-dynamics on the quantitative characteristics of individual structural groups. For studies aimed at characterizing CP-dynamics, a series of variable-contact-time experiments combined with kinetic modelling is recommended because it provides more comprehensive information than a single fixed contact time under the experimental conditions employed in the present study.

The methodological approach proposed here may extend the analytical applicability of CP/MAS ^13^C NMR spectroscopy for HAs and related heterogeneous natural organic materials by providing additional effective descriptors of CP-dynamics. In particular, CP-dynamic parameters derived from multipoint experiments may serve as complementary effective descriptors reflecting differences in structural heterogeneity and supramolecular organization of HAs.

Because the experiments were performed under conventional CP/MAS conditions without independent measurements of proton relaxation times, the observed differences in absolute signal intensity should be interpreted primarily in a comparative rather than strictly quantitative sense.

## Figures and Tables

**Table 1:** Effective CP-dynamic parameters obtained by nonlinear fitting of the classical three-parameter and extended four-parameter CP-dynamic models.

Molecular Fragments	Horizon, Depth, cm	Classical Three-Parameter Model		Extended Four-Parameter Model		
		*T_CH_*, ms	*T*_1*ρH*_, ms	*T_CH_*_1_, ms	*T_CH_*_2_, ms	*T*_1*ρH*_, ms
C,H-alkyl C	Hi 0–10	0.053 ± 0.004	4.4 ± 0.3	0.035 ± 0.008	0.13 ± 0.03	4.3 ± 0.3
	He 10–20	0.047 ± 0.003	2.91 ± 0.18	0.047 ± 0.014	0.070 ± 0.011	2.91 ± 0.16
	Hi1 20–30	0.048 ± 0.003	3.34 ± 0.21	0.034 ± 0.007	0.107 ± 0.023	3.26 ± 0.18
	Hi2 30–33	0.036 ± 0.004	2.48 ± 0.24	0.036 ± 0.007	0.054 ± 0.021	2.48 ± 0.20
	Hif 33–50	0.035 ± 0.003	2.64 ± 0.21	0.035 ± 0.011	0.053 ± 0.008	2.64 ± 0.21
	Hef1 50–70	0.036 ± 0.003	2.45 ± 0.16	0.036 ± 0.011	0.054 ± 0.009	2.45 ± 0.15
methoxyl/ N-alkyl C	Hi 0–10	0.043 ± 0.004	5.0 ± 0.5	0.030 ± 0.008	0.10 ± 0.03	4.8 ± 0.4
	He 10–20	0.038 ± 0.003	3.02 ± 0.21	0.038 ± 0.010	0.057 ± 0.010	3.02 ± 0.18
	Hi1 20–30	0.041 ± 0.004	3.3 ± 0.3	0.041 ± 0.008	0.062 ± 0.024	3.30 ± 0.23
	Hi2 30–33	0.040 ± 0.005	3.2 ± 0.3	0.040 ± 0.008	0.061 ± 0.028	3.2 ± 0.3
	Hif 33–50	0.035 ± 0.003	3.14 ± 0.26	0.035 ± 0.011	0.052 ± 0.009	3.14 ± 0.22
	Hef1 50–70	0.047 ± 0.003	3.29 ± 0.23	0.047 ± 0.015	0.070 ± 0.011	3.29 ± 0.21
O-alkyl C	Hi 0–10	0.041 ± 0.004	3.5 ± 0.3	0.041 ± 0.007	0.061 ± 0.023	3.5 ± 0.3
	He 10–20	0.033 ± 0.004	2.32 ± 0.27	0.033 ± 0.007	0.050 ± 0.023	2.32 ± 0.28
	Hi1 20–30	0.030 ± 0.003	2.95 ± 0.25	0.030 ± 0.010	0.045 ± 0.008	2.95 ± 0.27
	Hi2 30–33	0.033 ± 0.005	1.87 ± 0.23	0.033 ± 0.007	0.049 ± 0.026	1.87 ± 0.22
	Hif 33–50	0.034 ± 0.004	2.08 ± 0.22	0.034 ± 0.013	0.052 ± 0.010	2.08 ± 0.19
	Hef1 50–70	0.033 ± 0.004	2.35 ± 0.28	0.033 ± 0.007	0.049 ± 0.028	2.35 ± 0.24
aryl C	Hi 0–10	0.066 ± 0.011	8.0 ± 1.4	0.040 ± 0.006	1.6 ± 0.6	5.0 ± 0.4
	He 10–20	0.036 ± 0.003	5.9 ± 0.4	0.036 ± 0.006	0.053 ± 0.016	5.9 ± 0.4
	Hi1 20–30	0.047 ± 0.005	6.7 ± 0.8	0.047 ± 0.010	0.071 ± 0.028	6.7 ± 0.7
	Hi2 30–33	0.036 ± 0.004	5.2 ± 0.5	0.036 ± 0.007	0.055 ± 0.020	5.2 ± 0.5
	Hif 33–50	0.036 ± 0.004	6.1 ± 0.6	0.036 ± 0.007	0.054 ± 0.019	6.1 ± 0.5
	Hef1 50–70	0.046 ± 0.003	7.1 ± 0.5	0.038 ± 0.008	0.082 ± 0.021	7.1 ± 0.4
O,N-aryl C	Hi 0–10	0.33 ± 0.11	13 ± 5	0.10 ± 0.04	1.8 ± 1.8 *	12 ± 7
	He 10–20	0.15 ± 0.04	11 ± 3	0.056 ± 0.020	1.3 ± 1.8 *	8.1 ± 2.2
	Hi1 20–30	0.39 ± 0.11	6.9 ± 1.7	0.21 ± 0.07	2.0 ± 1.9	6.0 ± 1.0
	Hi2 30–33	0.22 ± 0.05	11 ± 4	0.13 ± 0.03	2.7 ± 2.9 *	7.6 ± 1.5
	Hif 33–50	0.30 ± 0.08	11 ± 3	0.11 ± 0.04	1.5 ± 1.6 *	9.5 ± 2.4
	Hef1 50–70	0.33 ± 0.09	10 ± 3	0.15 ± 0.06	1.7 ± 1.6	8.6 ± 2.1
carboxyl/ amide C	Hi 0–10	0.59 ± 0.07	5.9 ± 0.6	0.40 ± 0.06	1.8 ± 1.3	5.4 ± 0.6
	He 10–20	0.35 ± 0.06	5.1 ± 0.8	0.20 ± 0.05	1.6 ± 1.1	4.6 ± 0.7
	Hi1 20–30	0.45 ± 0.08	5.0 ± 0.8	0.28 ± 0.05	2.4 ± 2.2	4.4 ± 0.5
	Hi2 30–33	0.29 ± 0.05	4.6 ± 0.7	0.17 ± 0.03	1.6 ± 1.2	4.0 ± 0.5
	Hif 33–50	0.30 ± 0.08	5.5 ± 1.1	0.15 ± 0.04	1.9 ± 2.4 *	4.7 ± 0.7
	Hef1 50–70	0.31 ± 0.05	5.7 ± 0.9	0.18 ± 0.03	1.6 ± 1.2	4.9 ± 0.7

* Parameter not reliably estimable (standard error ≥ 100% of the estimate); reported only as an approximate effective descriptor and not used for inter-sample comparisons.

**Table 2:** Goodness-of-fit and model-selection statistics for the classical three-parameter and extended four-parameter CP-dynamic models.

Molecular Fragments	Horizon, Depth, cm	Classical Three-Parameter Model				Extended Four-Parameter Model			
		*R* ^2^	RMSE, a.u.	AIC	BIC	*R* ^2^	RMSE, a.u.	AIC	BIC
C,H-alkyl C	Hi 0–10	0.986	6339	272.5	274.8	0.987	6140	273.7	276.5
	He 10–20	0.993	3859	259.6	261.9	0.993	3859	261.6	264.4
	Hi1 20–30	0.992	4302	262.4	264.7	0.993	4193	263.8	266.6
	Hi2 30–33	0.982	3064	253.6	255.9	0.982	3064	255.6	258.4
	Hif 33–50	0.988	2977	252.9	255.1	0.988	2977	254.9	257.7
	Hef1 50–70	0.991	2271	245.8	248.1	0.991	2272	247.8	250.6
methoxyl/ N-alkyl C	Hi 0–10	0.977	3247	255.1	257.4	0.977	3214	256.9	259.7
	He 10–20	0.991	1799	239.8	242.0	0.991	1799	241.8	244.6
	Hi1 20–30	0.983	2449	247.8	250.0	0.983	2449	249.8	252.6
	Hi2 30–33	0.977	2791	251.2	253.4	0.977	2791	253.2	256.0
	Hif 33–50	0.986	1966	242.1	244.3	0.986	1966	244.1	246.9
	Hef1 50–70	0.990	1844	240.4	242.7	0.990	1844	242.4	245.2
O-alkyl C	Hi 0–10	0.985	5836	270.4	272.6	0.985	5836	272.4	275.2
	He 10–20	0.976	4285	262.3	264.6	0.976	4285	264.3	267.1
	Hi1 20–30	0.985	2323	246.4	248.7	0.985	2323	248.4	251.2
	Hi2 30–33	0.973	4367	262.8	265.1	0.973	4367	264.8	267.6
	Hif 33–50	0.980	3546	257.4	259.7	0.980	3546	259.4	262.2
	Hef1 50–70	0.973	3892	259.8	262.1	0.973	3892	261.8	264.7
aryl C	Hi 0–10	0.929	3650	258.2	260.4	0.985	1680	240.0	242.8
	He 10–20	0.984	1527	235.5	237.8	0.984	1527	237.5	240.3
	Hi1 20–30	0.966	2421	247.5	249.7	0.966	2421	249.5	252.3
	Hi2 30–33	0.973	1895	241.1	243.4	0.973	1895	243.1	245.9
	Hif 33–50	0.975	1827	240.2	242.4	0.975	1827	242.2	245.0
	Hef1 50–70	0.986	1428	233.8	236.0	0.986	1426	235.7	238.5
O,N-aryl C	Hi 0–10	0.809	3240	255.1	257.3	0.887	2484	250.2	253.0
	He 10–20	0.799	2252	245.6	247.9	0.907	1529	237.5	240.4
	Hi1 20–30	0.901	2269	245.8	248.1	0.923	1999	244.5	247.3
	Hi2 30–33	0.893	1995	242.4	244.7	0.937	1535	237.6	240.5
	Hif 33–50	0.836	2298	246.1	248.4	0.887	1902	243.2	246.0
	Hef1 50–70	0.883	2384	247.1	249.3	0.914	2042	245.1	247.9
carboxyl/ amide C	Hi 0–10	0.987	2628	249.6	251.9	0.989	2498	250.3	253.1
	He 10–20	0.958	2816	251.4	253.7	0.970	2382	249.1	251.9
	Hi1 20–30	0.965	2786	251.1	253.4	0.978	2198	247.0	249.8
	Hi2 30–33	0.971	2213	245.1	247.4	0.976	1724	240.7	243.5
	Hif 33–50	0.921	3010	253.1	255.4	0.958	2203	247.0	249.8
	Hef1 50–70	0.958	2404	247.3	249.6	0.974	1886	243.0	245.8

**Table 3:** Elemental composition of HA samples (adapted from [10]).

Horizon, Depth, cm	Mass Fraction, %				Ash, %
	C	N	H	O	
Hi, 0–10	56.0 ± 2.0	3.8 ± 0.4	5.6 ± 0.5	34.6 ± 2.1	0.23
He, 10–20	57.2 ± 2.0	4.0 ± 0.4	5.0 ± 0.5	33.8 ± 2.1	0.50
Hi1, 20–30	58.0 ± 2.0	4.0 ± 0.4	4.8 ± 0.4	33.2 ± 2.1	0.47
Hi2, 30–33	56.6 ± 2.0	3.3 ± 0.4	4.2 ± 0.4	36.0 ± 2.0	0.58
Hif, 33–50	56.6 ± 2.0	3.2 ± 0.4	4.2 ± 0.4	36.1 ± 2.0	0.59
Hef1, 50–70	58.0 ± 2.0	3.5 ± 0.4	4.3 ± 0.4	34.3 ± 2.1	0.72

## Data Availability

All data supporting the findings of this study are available within the article and its Supplementary Materials.

## Supplementary Materials

The following supporting information can be downloaded at: https://www.mdpi.com/article/10.3390/molecules31173044/s1. Figure S1: CP/MAS ^13^C NMR spectra of HA samples from various horizons of the Hemic Folic Cryic Histosol (with varying contact times): (a) Hi; (b) He; (c) Hi1; (d) Hi2; (e) Hif; and (f) Hef1; Figure S2: Experimental CP/MAS ^13^C NMR signal intensities and fitted curves from the classical three-parameter and extended four-parameter models as a function of contact time for HA samples: (a) C,H-alkyl C; (b) methoxyl/N-alkyl C; (c) O-alkyl C; (d) aryl C; (e) O,N-aryl C; (f) carboxyl/amide C; Figure S3: Bootstrap distributions (1000 resamples) of the fitted parameters from the extended four-parameter model for the CP/MAS ^13^C NMR signal intensities of HA samples: (a) C,H-alkyl C; (b) methoxyl/N-alkyl C; (c) O-alkyl C; (d) aryl C; (e) O,N-aryl C; (f) carboxyl/amide C; Figure S4: Correlation matrices of the fitted parameters for the classical three-parameter (1) and extended four-parameter (2) models obtained for the HA samples: (a) C,H-alkyl C; (b) methoxyl/N-alkyl C; (c) O-alkyl C; (d) aryl C; (e) O,N-aryl C; (f) carboxyl/amide C; Figure S5: Residuals of the classical three-parameter (1) and extended four-parameter (2) models fitted to the CP/MAS ^13^C NMR signal intensities as a function of contact time for HA samples: (a) C,H-alkyl C; (b) methoxyl/N-alkyl C; (c) O-alkyl C; (d) aryl C; (e) O,N-aryl C; (f) carboxyl/amide C; Table S1: Numerical values of the experimental CP/MAS ^13^C NMR signal intensities (a.u.) used for kinetic modelling; Code S1: R script for nonlinear least-squares fitting of the CP-dynamics models, bootstrap resampling, and statistical diagnostics. File name: CP_kinetics_fitting.R.
