# Supplementary material for: Contact-Time Dependence and Cross-Polarization Kinetics of Apparent Signal Distributions in CP/MAS 13C NMR Spectra of Humic Acids
Source: Molecules. 2026 Aug 30;31(17):3044. doi: 10.3390/molecules31173044 (PMC13567671; doi:10.3390/molecules31173044)
Supplement: Supplementary file 1 [file molecules-31-03044-s001.zip › suppelmentary materials.pdf]

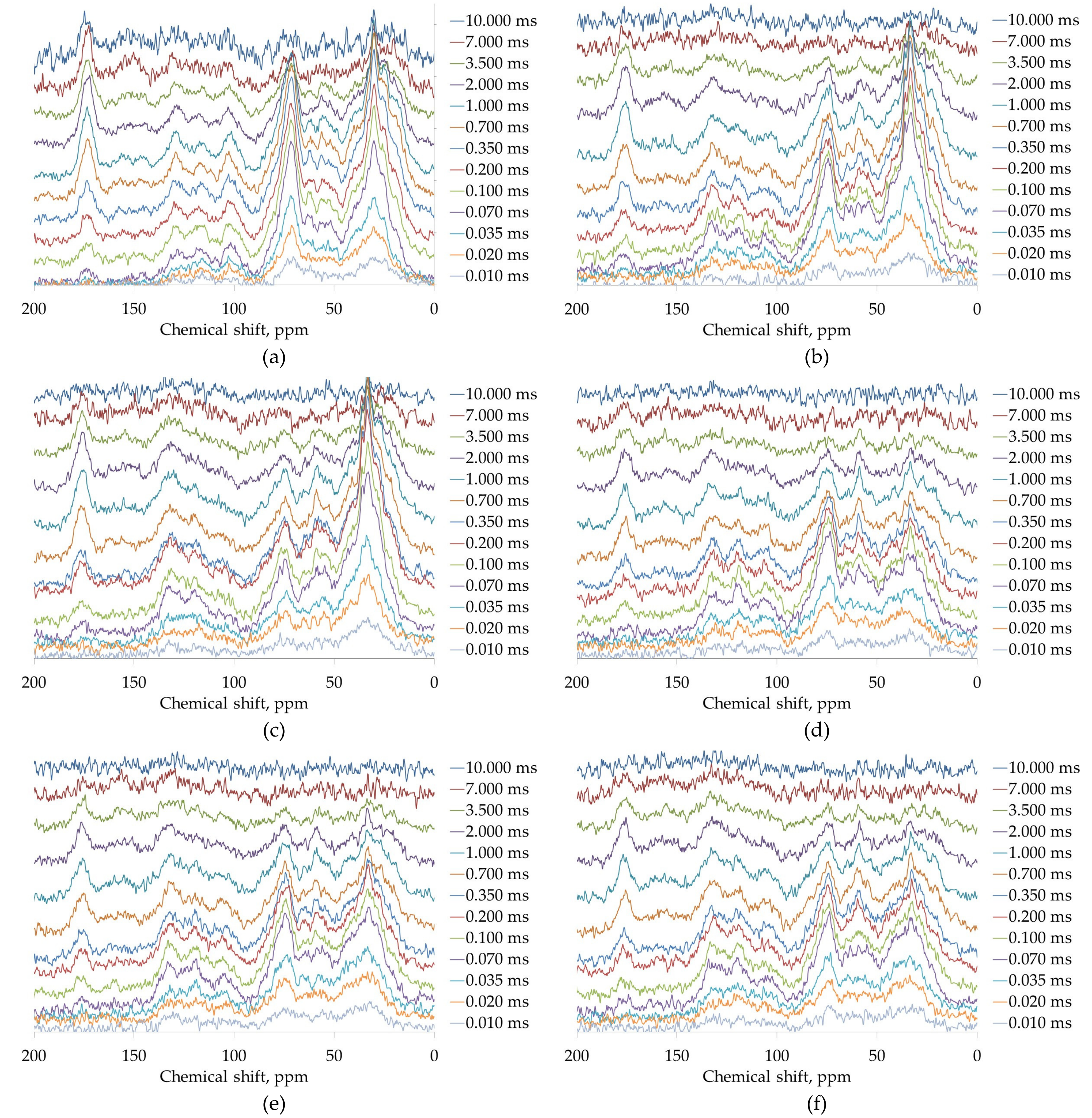

Figure S1. CP/MAS ^{13}C NMR spectra of HA samples from various horizons of the Hemic Folic Cryic Histosol (with varying contact times): (a) Hi; (b) He; (c) Hi1; (d) Hi2; (e) Hif; and (f) Hef1.

Table S1. Numerical values of the experimental CP/MAS ^{13}C NMR signal intensities (a.u.) used for kinetic modelling.

Molecular fragments	Layer, depth, cm	Contact time, ms												
		**0.010**	**0.020**	**0.035**	**0.070**	**0.100**	**0.200**	**0.350**	**0.700**	**1.000**	**2.000**	**3.500**	**7.000**	**10.000**
C,H-alkyl C	Hi 0–10	26,862	56,173	84,377	138,651	152,288	153,107	166,417	162,350	154,483	115,954	81,569	33,521	17,715
	He 10–20	25,097	48,955	76,551	122,101	132,762	131,115	136,673	120,960	110,148	73,815	45,680	13,607	6667
	Hi1 20–30	28,538	55,576	82,255	126,643	137,731	140,300	145,614	140,527	124,385	87,305	54,739	17,840	9334
	Hi2 30–33	19,896	27,607	41,678	64,158	73,182	63,603	63,370	53,151	50,604	33,120	17,621	5427	4872
	Hif 33–50	37,124	53,363	79,512	81,498	84,304	77,376	66,829	54,573	43,250	23,439	10,889	5750	37124
	Hef1 50–70	34,540	46,339	71,620	72,021	73,086	69,309	62,172	53,289	33,352	19,886	5673	6378	34,540
methoxyl/ N-alkyl C	Hi 0–10	13,351	27,412	39,264	60,846	63,140	61,995	64,876	67,204	63,042	52,378	34,924	13,636	9182
	He 10–20	10,721	24,153	36,294	53,289	55,390	55,158	51,942	50,150	44,916	29,554	19,823	7005	640
	Hi1 20–30	13,416	23,652	31,783	54,783	56,461	54,970	53,716	54,228	47,718	34,808	19,640	6748	4491
	Hi2 30–33	12,622	21,490	31,744	56,184	52,113	52,713	55,826	47,873	46,934	30,813	20,564	6696	4594
	Hif 33–50	23,927	32,076	48,627	49,958	47,516	46,772	41,410	41,137	29,207	15,266	6226	3760	23,927
	Hef1 50–70	21,127	30,530	50,360	49,626	58,011	53,495	50,811	44,177	33,510	18,881	8885	5096	21,127
O-alkyl C	Hi 0–10	27,405	57,534	85,763	138,122	138,670	134,425	139,714	130,539	123,166	92,267	52,896	20,034	8244
	He 10–20	17,826	37,686	52,859	87,218	82,818	77,341	74,933	61,592	58,285	38,340	19,954	7029	4579
	Hi1 20–30	16,449	27,802	40,118	60,130	56,860	55,035	53,483	49,036	45,009	27,785	19,847	7148	4153
	Hi2 30–33	18,534	33,819	51,598	81,423	80,552	71,296	68,719	53,648	45,742	32,573	14,857	5931	3586
	Hif 33–50	32,371	47,246	74,946	73,996	67,285	66,912	55,939	44,609	30,918	17,178	5852	2047	32,371
	Hef1 50–70	30,413	48,786	76,027	70,468	68,704	66,518	55,570	45,427	32,354	20,385	8237	4030	30,413
aryl C	Hi 0–10	9454	14,041	19,168	34,516	36,970	40,234	40,372	47,820	48,115	43,090	35,227	16,690	11,646
	He 10–20	8372	17,246	26,748	39,634	39,631	41,978	38,030	39,199	35,581	31,321	21,542	13,452	9057
	Hi1 20–30	11,452	14,062	22,868	39,822	41,073	42,756	41,589	42,917	44,180	37,859	30,465	14,276	9380
	Hi2 30–33	8463	18,746	22,953	35,510	38,569	39,270	37,693	34,324	32,483	31,960	17,908	9630	8259
	Hif 33–50	16,494	24,035	37,009	42,155	39,254	37,138	40,319	36,539	31,205	22,296	13,439	8879	16,494
	Hef1 50–70	16,789	22,280	37,044	39,241	44,802	41,922	41,108	40,901	37,032	26,956	15,569	12,215	16,789
O,N-aryl C	Hi 0–10	5267	5836	5050	11,293	10,846	12,806	14,623	20,442	25,277	26,224	25,534	18,004	10,675
	He 10–20	1738	6520	7345	7213	9096	11,059	14,412	15,845	16,743	19,918	14,823	8775	6010
	Hi1 20–30	4776	1370	2112	7429	8008	13,764	13,365	18,812	23,051	22,050	20,047	7375	7475
	Hi2 30–33	4028	4559	4703	6979	8430	13,137	15,378	18,940	20,559	17,807	20,964	12,591	6839
	Hif 33–50	3543	5037	7447	7929	11,543	11,452	17,633	20,186	20,668	16,851	13,054	7064	3543
	Hef1 50–70	3293	1298	8048	8184	15,013	11,053	19,294	21,478	22,475	19,865	10,808	9683	3293
carboxyl/ amide C	Hi 0–10	4157	6435	8281	16,300	20,709	27,582	40,369	60,583	71,111	71,075	55,978	32,040	17,203
	He 10–20	4035	3562	6805	14,081	18,988	22,821	29,449	37,776	45,552	39,442	29,308	11,528	8621
	Hi1 20–30	3831	5502	5162	13,440	16,292	24,658	28,181	41,894	44,171	44,974	34,375	15,043	6129
	Hi2 30–33	4270	5345	6214	10,626	16,656	20,804	25,406	33,984	34,058	32,169	20,608	10,088	2491
	Hif 33–50	6641	9611	11,289	14,999	19,979	23,385	31,325	32,946	32,512	26,418	9619	4177	6641
	Hef1 50–70	5355	3353	12,843	13,849	23,115	25,106	33,623	35,906	36,041	24,531	11,435	7752	5355

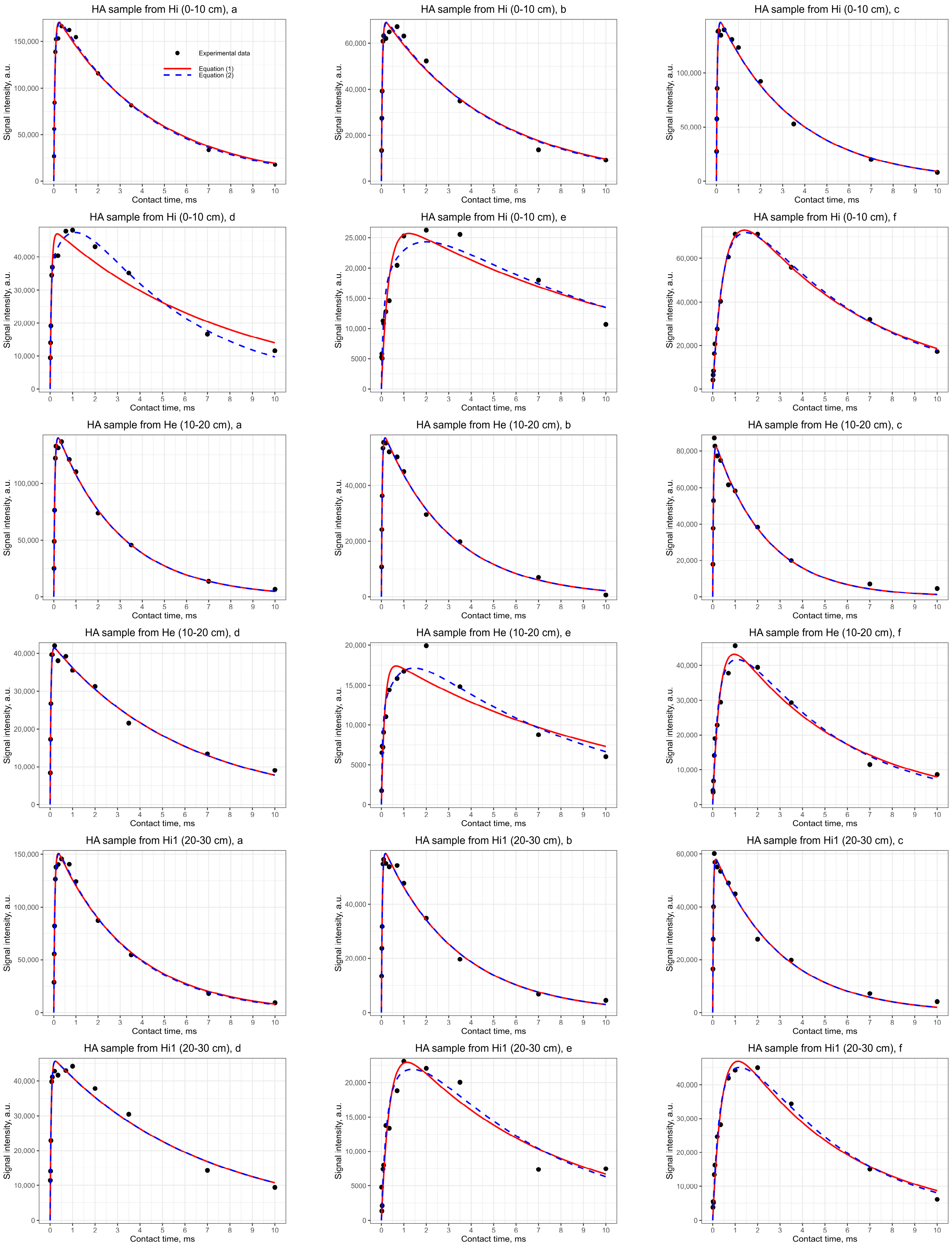

HA sample from Hi (0-10 cm), a
HA sample from Hi (0-10 cm), b
HA sample from Hi (0-10 cm), c
HA sample from Hi (0-10 cm), d
HA sample from Hi (0-10 cm), e
HA sample from Hi (0-10 cm), f
HA sample from He (10-20 cm), a
HA sample from He (10-20 cm), b
HA sample from He (10-20 cm), c
HA sample from He (10-20 cm), d
HA sample from He (10-20 cm), e
HA sample from He (10-20 cm), f
HA sample from Hi1 (20-30 cm), a
HA sample from Hi1 (20-30 cm), b
HA sample from Hi1 (20-30 cm), c
HA sample from Hi1 (20-30 cm), d
HA sample from Hi1 (20-30 cm), e
HA sample from Hi1 (20-30 cm), f
Experimental data
Equation (1)
Equation (2)
Signal intensity, a.u.
Contact time, ms

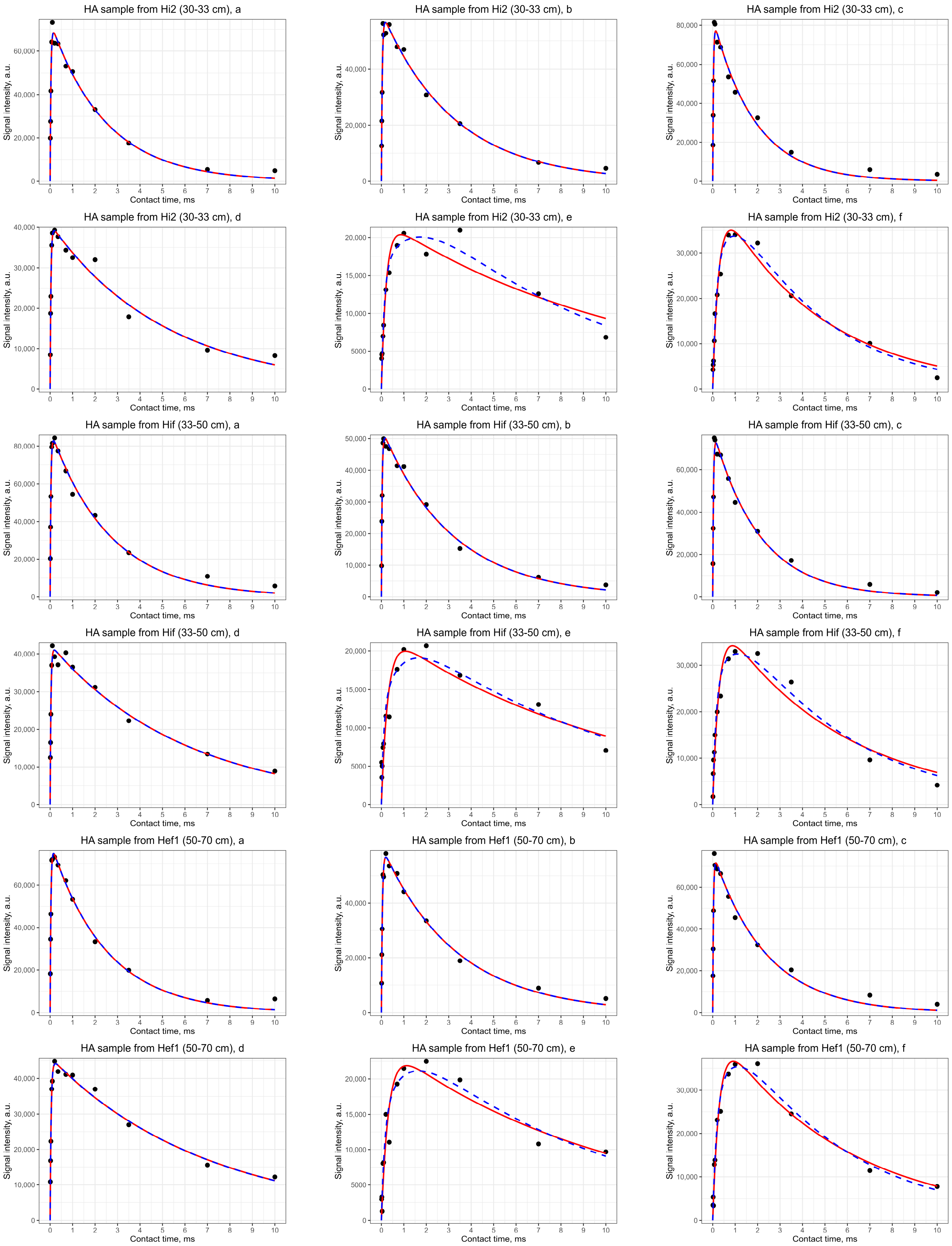

Figure S2. Experimental CP/MAS ^{13}C NMR signal intensities and fitted curves from the classical three-parameter and extended four-parameter models as a function of contact time for HA samples: (a) C,H-alkyl C; (b) methoxyl/N-alkyl C; (c) O-alkyl C; (d) aryl C; (e) O,N-aryl C; (f) carboxyl/amide C.

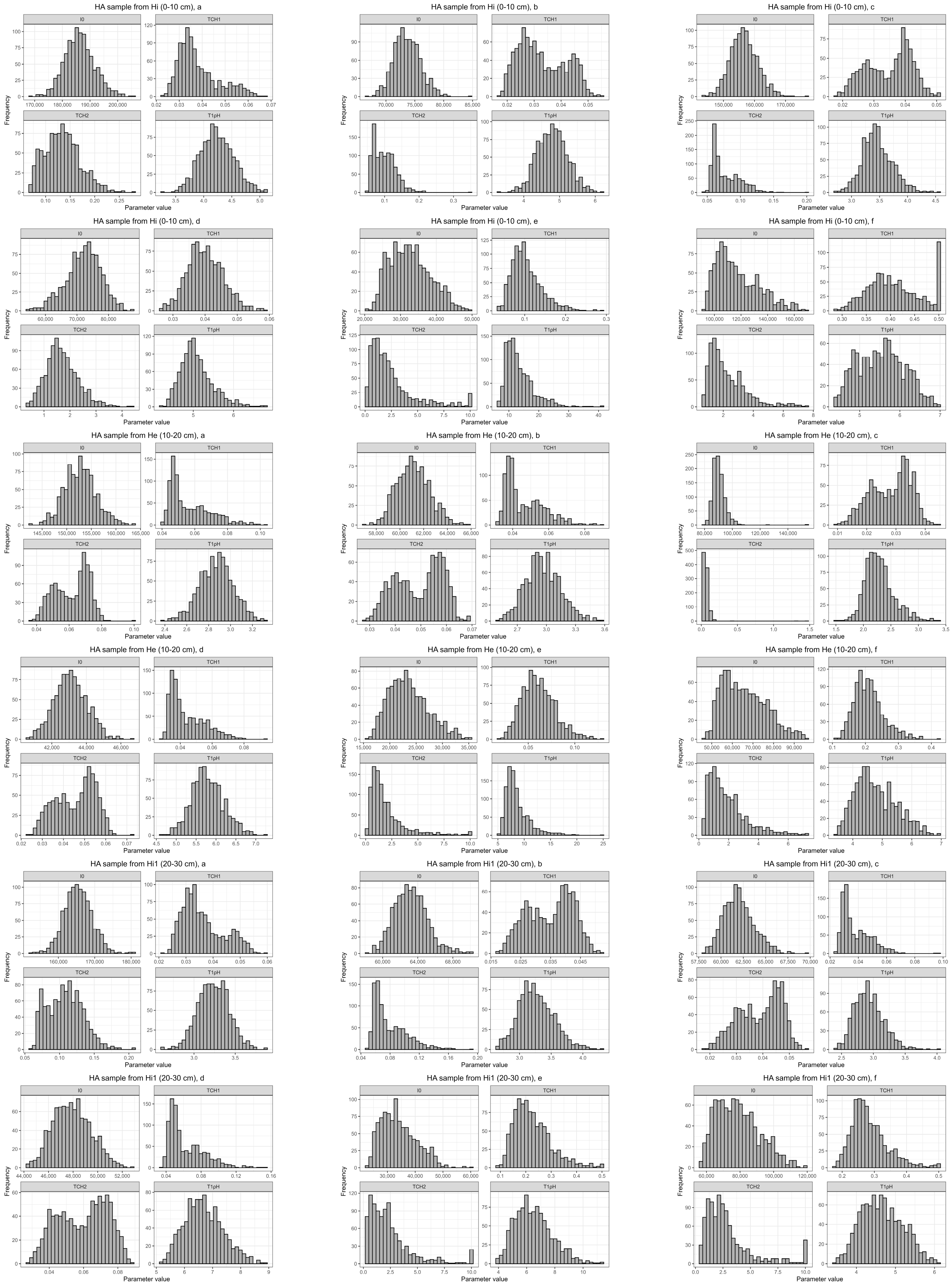
HA sample from Hi (0-10 cm), a
HA sample from Hi (0-10 cm), b
HA sample from Hi (0-10 cm), c
HA sample from Hi (0-10 cm), d
HA sample from Hi (0-10 cm), e
HA sample from Hi (0-10 cm), f
HA sample from He (10-20 cm), a
HA sample from He (10-20 cm), b
HA sample from He (10-20 cm), c
HA sample from He (10-20 cm), d
HA sample from He (10-20 cm), e
HA sample from He (10-20 cm), f
HA sample from Hi1 (20-30 cm), a
HA sample from Hi1 (20-30 cm), b
HA sample from Hi1 (20-30 cm), c
HA sample from Hi1 (20-30 cm), d
HA sample from Hi1 (20-30 cm), e
HA sample from Hi1 (20-30 cm), f
I0
TCH1
TCH2
T1pH
Frequency
Parameter value

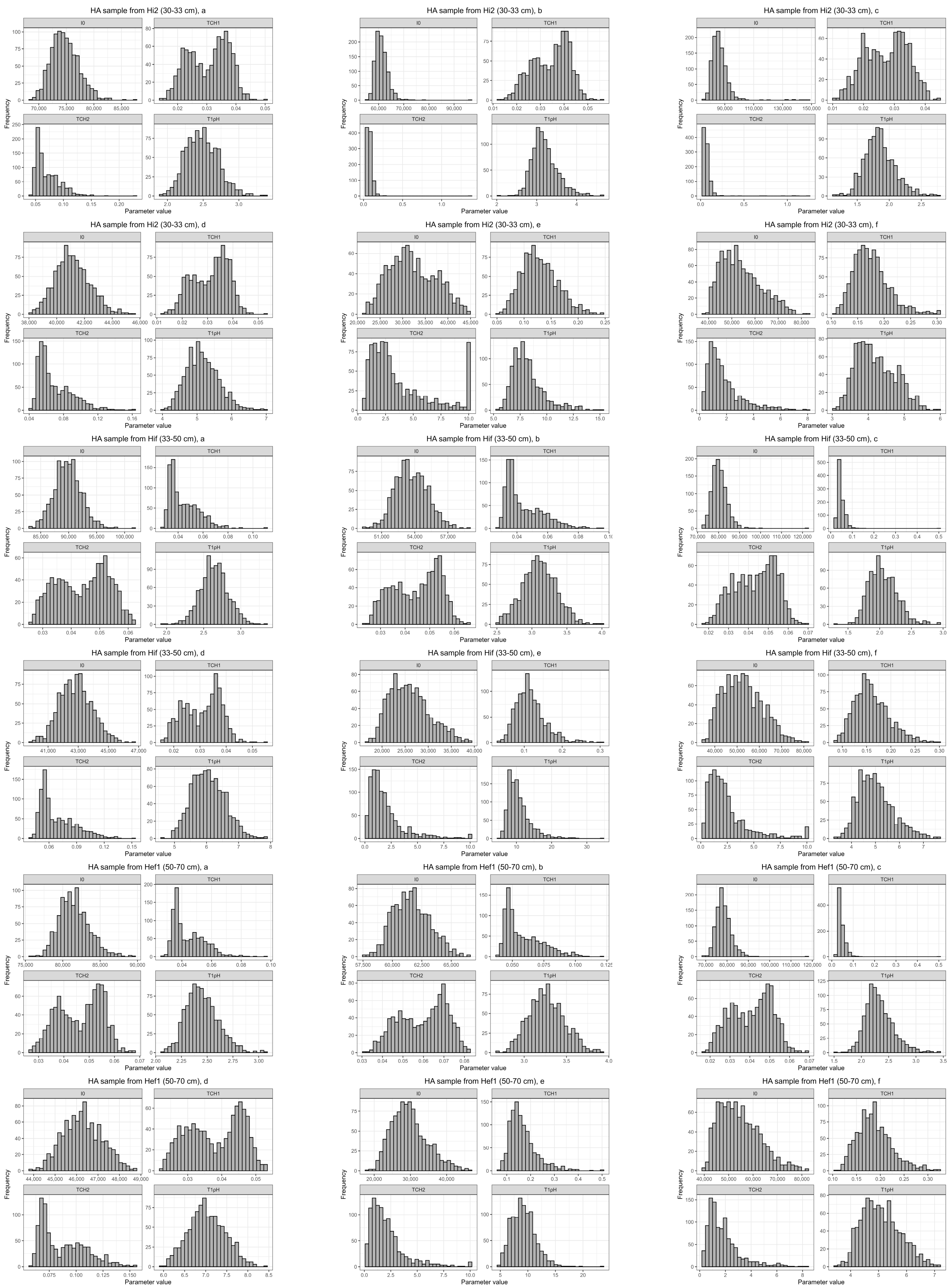

Figure S3. Bootstrap distributions (1000 resamples) of the fitted parameters from the extended four-parameter model for the CP/MAS ^{13}C NMR signal intensities of HA samples: (a) C,H-alkyl C; (b) methoxyl/N-alkyl C; (c) O-alkyl C; (d) aryl C; (e) O,N-aryl C; (f) carboxyl/amide C.

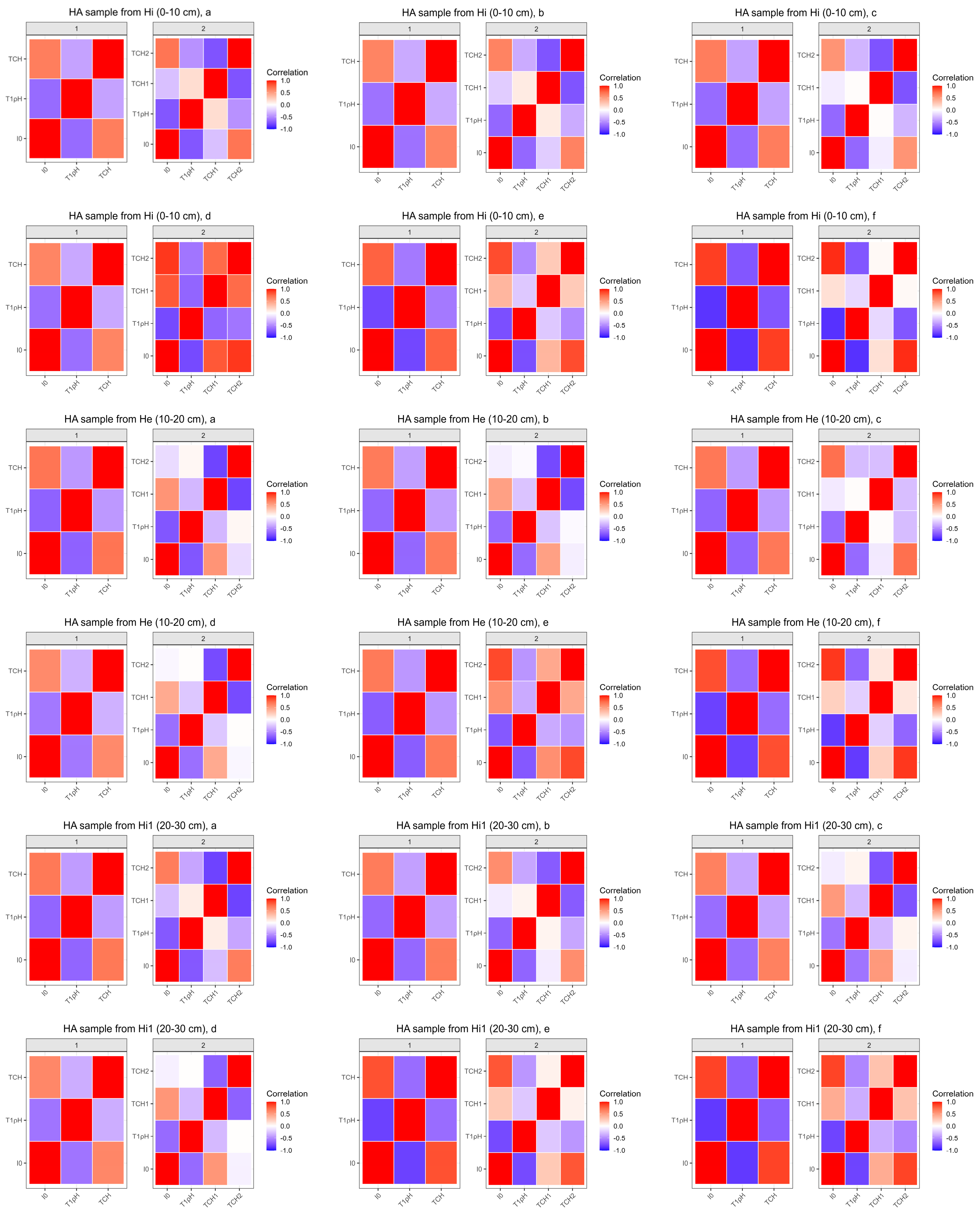
HA sample from Hi (0-10 cm), a
HA sample from Hi (0-10 cm), b
HA sample from Hi (0-10 cm), c
HA sample from Hi (0-10 cm), d
HA sample from Hi (0-10 cm), e
HA sample from Hi (0-10 cm), f
HA sample from He (10-20 cm), a
HA sample from He (10-20 cm), b
HA sample from He (10-20 cm), c
HA sample from He (10-20 cm), d
HA sample from He (10-20 cm), e
HA sample from He (10-20 cm), f
HA sample from Hi1 (20-30 cm), a
HA sample from Hi1 (20-30 cm), b
HA sample from Hi1 (20-30 cm), c
HA sample from Hi1 (20-30 cm), d
HA sample from Hi1 (20-30 cm), e
HA sample from Hi1 (20-30 cm), f
1
2
TCH
T1pH
I0
TCH2
TCH1
Correlation
1.0
0.5
0.0
-0.5
-1.0

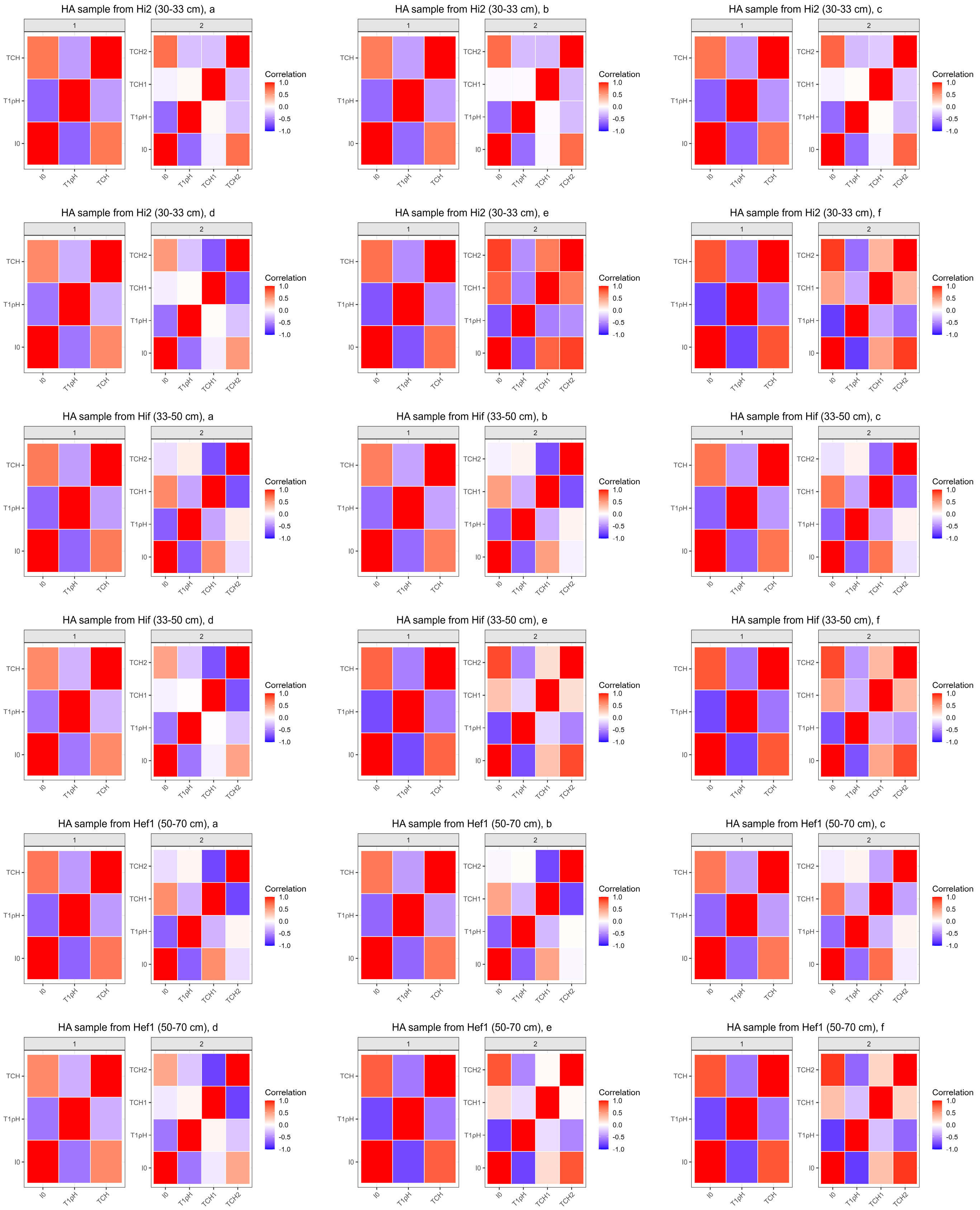

Figure S4. Correlation matrices of the fitted parameters for the classical three-parameter (1) and extended four-parameter (2) models obtained for the HA samples: (a) C,H-alkyl C; (b) methoxyl/N-alkyl C; (c) O-alkyl C; (d) aryl C; (e) O,N-aryl C; (f) carboxyl/amide C.

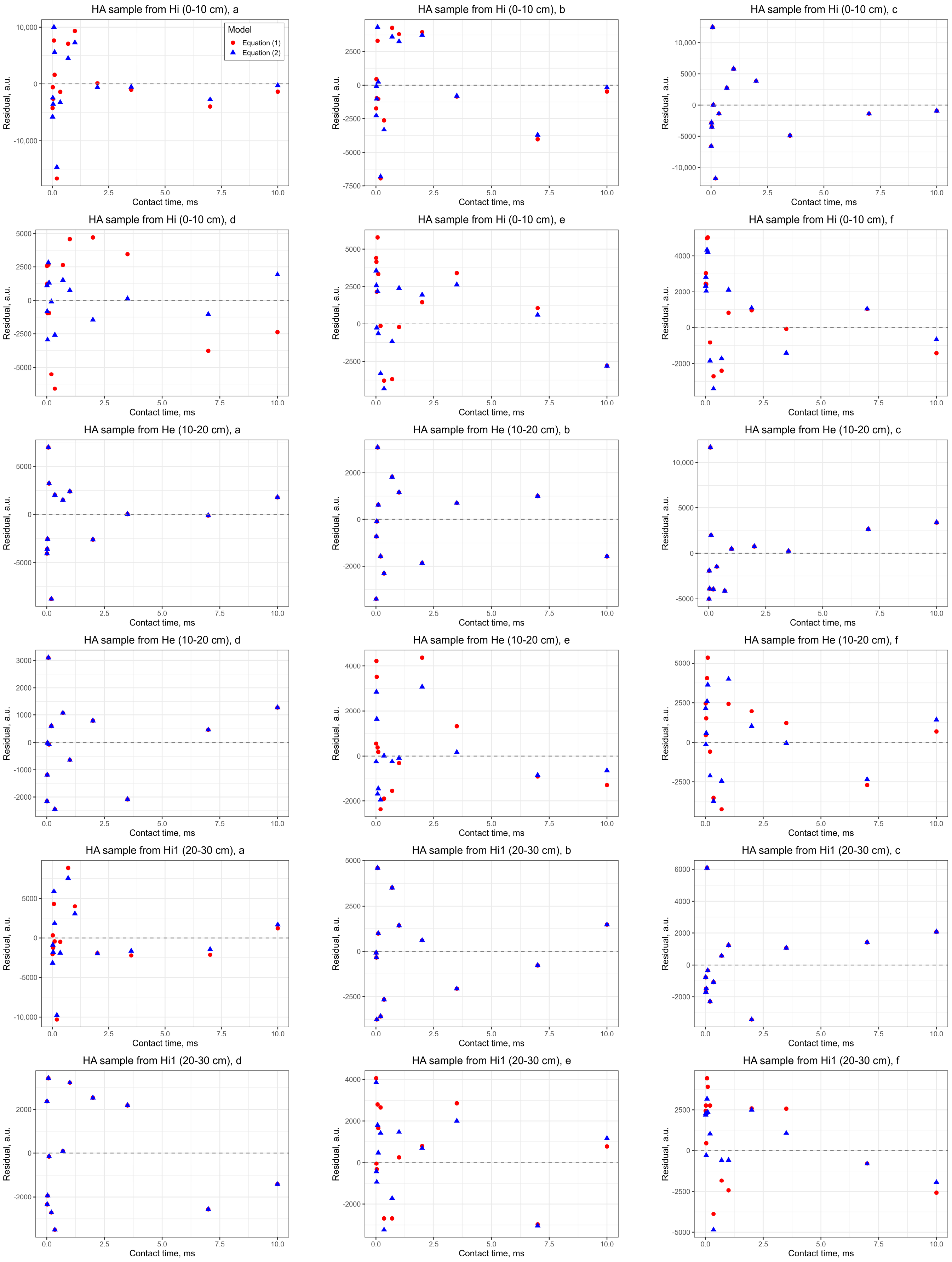
HA sample from Hi (0-10 cm), a
HA sample from Hi (0-10 cm), b
HA sample from Hi (0-10 cm), c
HA sample from Hi (0-10 cm), d
HA sample from Hi (0-10 cm), e
HA sample from Hi (0-10 cm), f
HA sample from He (10-20 cm), a
HA sample from He (10-20 cm), b
HA sample from He (10-20 cm), c
HA sample from He (10-20 cm), d
HA sample from He (10-20 cm), e
HA sample from He (10-20 cm), f
HA sample from Hi1 (20-30 cm), a
HA sample from Hi1 (20-30 cm), b
HA sample from Hi1 (20-30 cm), c
HA sample from Hi1 (20-30 cm), d
HA sample from Hi1 (20-30 cm), e
HA sample from Hi1 (20-30 cm), f
Model
Equation (1)
Equation (2)
Residual, a.u.
Contact time, ms

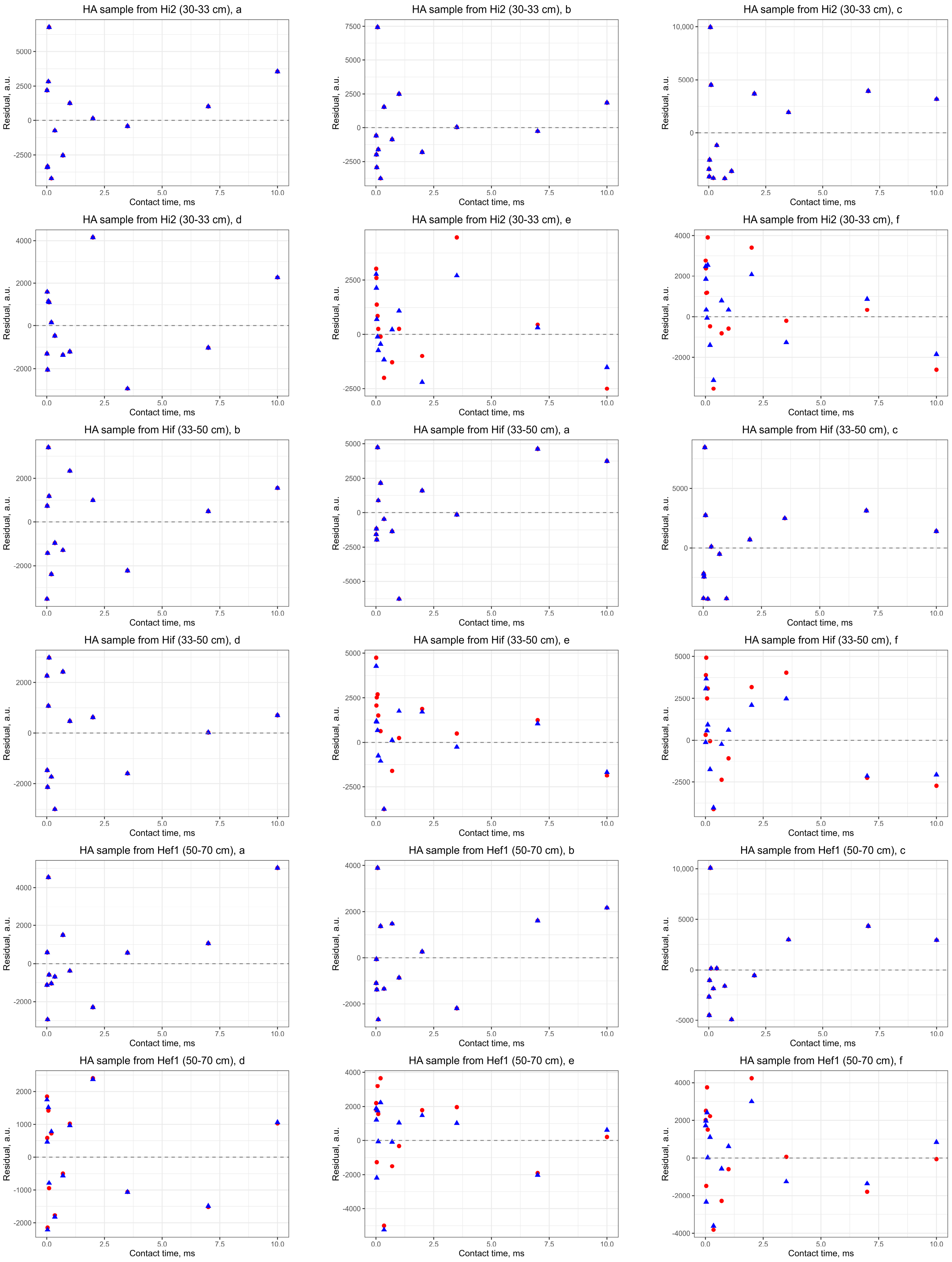

Figure S5. Residuals of the classical three-parameter (**1**) and extended four-parameter (2) models fitted to the CP/MAS ^{13}C NMR signal intensities as a function of contact time for HA samples: (a) C,H-alkyl C; (b) methoxyl/N-alkyl C; (c) O-alkyl C; (d) aryl C; (e) O,N-aryl C; (f) carboxyl/amide C.
